# Antenatal interventions to reduce preterm birth: an overview of Cochrane systematic reviews

**DOI:** 10.1186/1756-0500-7-265

**Published:** 2014-04-23

**Authors:** Brigitte Piso, Ingrid Zechmeister-Koss, Roman Winkler

**Affiliations:** 1 Ludwig Boltzmann Institute for Health Technology Assessment, Garnisongasse 7/20, 1090 Wien, Austria

**Keywords:** Premature birth, Maternity and child health, Pregnancy

## Abstract

**Background:**

Several factors are associated with an increased risk of preterm birth (PTB); therefore, various interventions might have the potential to influence it. Due to the large number of interventions that address PTB, the objective of this overview is to summarise evidence from Cochrane reviews regarding the effects and safety of these different interventions.

**Methods:**

We conducted a systematic literature search in the Cochrane Database of Systematic Reviews. Included reviews should be based on randomised controlled trials comparing antenatal non-pharmacological and pharmacological interventions that directly or indirectly address PTB with placebo/no treatment or routine care in pregnant women at less than 37 completed weeks of gestation without signs of threatened preterm labour. We considered PTB at less than 37 completed weeks of gestation as the primary outcome.

**Results:**

We included 56 Cochrane systematic reviews. Three interventions increased PTB risk significantly. Twelve interventions led to a statistically significant lower incidence of PTBs. However, this reduction was mostly observed in defined at-risk subgroups of pregnant women. The remaining antenatal interventions failed to prove a significant effect on PTB < 37 weeks, but some of them at least showed a positive effect in secondary outcomes (e.g., reduction in early PTBs). As an unintended result of this review, we identified 28 additional Cochrane reviews which intended to report on PTB < 37 weeks, but were not able to find any RCTs reporting appropriate data.

**Conclusions:**

The possible effects of a diverse range of interventions on PTB have been evaluated in Cochrane systematic reviews. Few interventions have been demonstrated to be effective and a small number have been found to be harmful. For around half of the interventions evaluated, the Cochrane review concluded that there was insufficient evidence to provide sound recommendations for clinical practice. No RCT evidence is available for a number of potentially relevant interventions.

## Background

Preterm birth (PTB) is defined as childbirth occurring at less than 37 completed weeks of gestation [1]. PTB is a major determinant of neonatal mortality and morbidity, and has long-term, adverse consequences for health (e.g., learning disabilities or visual and hearing problems). On average, 12% of babies are born too soon in the poorest countries, compared with 9% in higher-income countries [2]. In almost all countries with reliable data, PTB rates are increasing. Possible reasons for this include increases in maternal age and underlying maternal health problems such as diabetes and hypertensive disorders, as well as iatrogenic factors like greater use of infertility treatments leading to increased rates of multiple pregnancies, and changes in obstetric practices such as more caesarean births before term [2].

PTB is a multi-factorial disorder. A wide spectrum of predisposing factors is associated with PTB [3,4]: There is a genetic influence and PTB rates differ across ethnic groups. Social stress and maternal factors (e.g., smoking, alcohol consumption, poor nutritional status, advanced maternal age) and several non-genital tract infections have also been shown to be associated with increased risk of PTB. Last but not least, intrauterine infection may contribute to 25-40% of PTBs. However, the causes of approximately half of the PTB cases are unknown.

Due to the number of different factors that might contribute to an increased PTB risk, several interventions might have the potential to influence it. According to a summary published in 2008 [5] on interventions which might be able to influence PTB risk, primary preventive measures like protein and calorie supplementation, calcium, vitamin C or E supplementation, as well as periodontal care failed to prove beneficial effects on PTB in randomised controlled trials. Among the screening measures for low-risk women, screening for and treatment of asymptomatic bacteriuria had been reported to reduce the PTB rate, while other screening and subsequent treatment measures (e.g., for/of ureaplasma urealyticum, group B streptococcus, trichomonas vaginalis and fetal fibronectin testing with subsequent metronidazole and erythromycin treatment) did not reduce PTBs. Among secondary preventive measures targeted at women with known risk factors for PTB, only low-dose aspirin had shown a reduction in PTBs for women with risk of pre-eclampsia, while calcium supplementation did not alter PTB rates, and antioxidants even showed an increased risk for PTB. Effects of bed rest, limited work and reduced sexual activity for women with increased PTB risk had not been researched at all. Omega-3 supplements had shown a significant PTB reduction in women at risk of PTB, while more intensive antenatal care had failed to prove significant effects on PTB. Though antibiotic treatment of bacterial vaginosis had preliminarily shown positive effects in subgroups of women with increased PTB risk, other trials displayed conflicting results (even an increase in PTBs for metronidazole treatment). Progesterone had been beneficial in some at-risk populations, while cerclage had shown a reduction in PTBs in women with a short cervix and a history of previous PTB, no reduction in women with a short cervix without a history of previous PTB, and an increase in women with twin pregnancy. Besides these primary and secondary preventive measures during pregnancy, preconceptional interventions for all women of reproductive age, as well as interventions targeted at women with immediate PTB risk, have been discussed as influencing actual PTB rates.

Given the large number of Cochrane reviews that address primary and secondary preventive antenatal interventions that might be able to modify PTB incidence, the objective of this overview is to summarise evidence from Cochrane reviews regarding their effects and safety. Hence, we pursued the following research question: In pregnant women at less than 37 completed weeks of gestation without signs of threatened preterm labour do non-pharmacological or pharmacological preventive, therapeutic or screening interventions, compared to no treatment, placebo or routine care (as stated by review authors), reduce the rate of preterm births prior to 37 completed weeks of gestation?

## Methods

### Literature search

We conducted a systematic literature search in the Cochrane Database of Systematic Reviews on the 28th of August 2012. The search strategy is elucidated in Additional file 1. We also searched in references of included reviews and on the Cochrane Library website for further reviews. We neither searched for reviews other than Cochrane systematic reviews and any primary research studies, nor did we contact any review or single trial author. Two overview authors independently assessed all the potential systematic reviews. Any disagreement was resolved through discussion or, if required, we consulted a third person.

### Eligibility criteria for considering reviews for inclusion

We included any published Cochrane systematic review of (individual or cluster) randomised controlled trials focusing on primary or secondary preventive antenatal interventions to directly or indirectly reduce PTBs. The participants in the reviews considered are pregnant women at less than 37 completed weeks of gestation without signs of threatened preterm labour (premature labour, premature rupture of membranes). These women include those of different risk groups for PTB or underlying conditions. If the population considered in the review also comprised women in preterm labour, we only extracted data of subgroup analyses for women without (threatened) preterm labour.

We included reviews that compared interventions with placebo/no treatment or ‘routine care’ (as defined by trialists). We included reviews of trials that analysed interventions used in various settings (inpatient, outpatient, home-based). As far as possible, we focused on interventions that were mainly studied in high-income countries, but we did not exclude reviews based on studies carried out in mixed populations (high- and low-income countries).

We excluded Cochrane reviews analysing effects of preconceptional care, tertiary prevention of PTB and treatments of diseases either uncommon in high-income countries or without apparent relation to PTB (for example, HIV, sickle cell anaemia or tuberculosis). We further excluded Cochrane reviews that exclusively compared different treatments or different regimens of one treatment (e.g., by dose or route of administration), or that reported effects of inevitable treatments for morbidities in pregnancy (e.g., eclampsia, gonorrhoea). Finally, we excluded Cochrane reviews that were not able to report on PTB rates due to the lack of primary research data or if the reviews’ findings were solely based on RCTs included in a more recent review.

### Types of outcome measures

We defined PTB less than 37 weeks of gestation as the primary outcome. Therefore, in this overview we only included reviews that considered PTB (sometimes also stated as ‘premature birth’, ‘gestational age at birth’ or ‘preterm labour’) as a predefined outcome.

As secondary outcomes; we analysed whether very preterm birth (PTB less than 34 to32 weeks), low birth weight (LBW, birth weight less than 2500 grams), small for gestational age (SGA, birth weight below the 10th percentile of gestational age), admissions to neonatal intensive care units (NICU, also defined in some reviews as ‘specialised neonatal care units’), stillbirths, miscarriages/perinatal deaths or complications due to interventions for mother or child (as defined by trialists) were influenced by the intervention in a statistically significant manner.

### Assessment of the methodological quality of included reviews

We did not reassess the eligibility criteria for inclusion of RCTs in the systematic reviews or the risk of bias of included primary research studies.

Due to Cochrane’s publishing policy (all systematic reviews have to follow methods according to the *Cochrane Handbook of Systematic Reviews of Interventions*[6]), we also refrained from a quality assessment of the included Cochrane reviews.

### Data extraction and management

A single researcher extracted the data from the reviews using a predefined data extraction form. A second researcher independently double-checked the data for correctness and completeness.

### Data synthesis

Because of the heterogeneity of interventions and the research design, we did not perform a meta-analysis to estimate a pooled effect size of results. Therefore, our findings are solely presented descriptively. We list the review characteristics in [see Additional file 2: Table S2]. For the main outcome – the effect estimations of interventions on PTB prior to 37 weeks of gestation – we have provided Additional file 3: Table S1, which include forest plots of pooled effect sizes reported in the single systematic reviews. For risk/odds ratios, including the 95% confidence intervals and an overview of the statistical significance of secondary outcomes, see [Additional file 4: Table S3].

We structured the presentation of results based on the following classification: First, we provide information on effects of various antenatal interventions from Cochrane reviews that primarily intended to report on the impact on preterm delivery (reviews entitled ‘preventing preterm birth/ delivery/miscarriage/labour’, Additional file 3: Table S1a). Under the heading ‘Ultrasound screening’ (Additional file 3: Table S1b), we present results of Cochrane reviews analysing the effects of ultrasound screening in pregnancy. In Additional file 3: Table S1c (‘Prevention, detection and management of infection’) we summarise results of Cochrane reviews for infection screenings and antimicrobial treatments. In Additional file 3: Table S1d (‘Prevention, detection and management of hypertension/ pre-eclampsia and hyperglycaemia/ [gestational] diabetes’) we present results of Cochrane reviews that primarily analysed various interventions with respect to effects on the mentioned conditions. Additional file 3: Table S1e (‘Nutritional supplements and dietary interventions’) provides information about effects on PTB of diverse supplements as well as other changes in dietary composition (of reviews entitled ‘in/during/for pregnancy’; Additional file 3: Table S1e). Additional file 3: Table S1f (‘Psychological interventions and alternative models of care’) summarises results of Cochrane reviews on organisational aspects of antenatal care. Finally, in Additional file 3: Table S1g (‘Prevention and management of other morbidities’) we summarise effects of various interventions that could not be allocated to one of the above mentioned categories (e.g., treatment of other specific morbidities or lifestyle modification).

## Results

### Study selection

**Figure 1:**
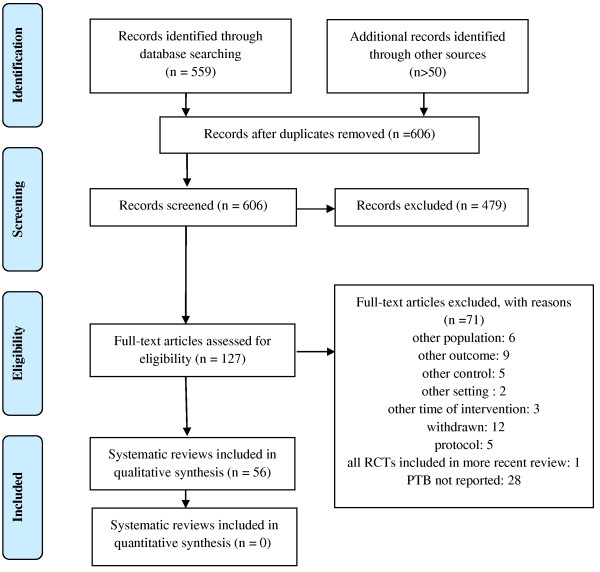
Literature selection process (PRISMA flow diagram).

The systematic literature search yielded 559 publications. After adding hand search results and subsequent de-duplication, we screened 606 citations on the abstract level and finally assessed 126 full text articles for eligibility. Of these, we included 56 Cochrane systematic reviews that met the inclusion criteria for this overview (Figure 1).

### Characteristics of included reviews

13 reviews addressed mechanical [7] or pharmaceutical prevention of PTB or miscarriage [8-12], the impact of other interventions targeted at influencing PTB or miscarriage [13-15], or analysed screening/risk assessment interventions to predict PTB risk and improve pregnancy outcomes [16-19]; two reviews analysed the effect of ultrasound screening on pregnancy outcomes [20,21] (Additional file 3: Table S1b). Seven reviews either addressed the prevention [22,23], the detection [24] or the treatment of infection [25-28] (Additional file 3: Table S1c). Twelve reviews analysed effects of various interventions applied for the prevention [29-36] or the treatment [37-40] of hypertensive disorders/pre-eclampsia; one review focused on interventions for pregnant women with hyperglycaemia [41] (Additional file 3: Table S1d). Eleven reviews covered general effects of dietary interventions on pregnancy outcomes [42-52] (Additional file 3: Table S1e). Four reviews analysed the effects of providing additional support [53] or of alternative models of care [54-56] (Additional file 3: Table S1f). The remaining six reviews addressed the prevention or management of other morbidities [57-62] (Additional file 3: Table S1g).

There was a great difference in the number of included RCTs (1–71) and women (45–25740). Diversity was also seen in the target population of the reviews (e.g., all pregnant women, women at risk of PTB or other predefined risk factors, women with or without a defined pre-existing morbidity, or women with multiple pregnancies). 18 reviews are based on trials all published before the year 2000. On the other hand, three reviews only included trials published within the last twelve years. The widest range of the publishing period is covered by the review on vitamin A supplementation in pregnancy (1931 to 2010) [44]. A more detailed description of the reviews’ characteristics can be found in [see Additional file 2: Table S2].

### Effects of interventions

#### a) Prevention of PTB or miscarriage and detection of PTB risk

Three interventions (cerclage with or without bed rest in women with singleton pregnancies at high risk of pregnancy loss [7], administration of progesterone in women with previous spontaneous PTB [8] and knowledge of fetal fibronectin test (FFT) [18]) showed a statistically significant PTB reduction, while one intervention (diethylstilbestrol supplementation) increased PTB risk [10] (Additional file 3: Table S1a). Additional positive effects in secondary outcomes were seen for cerclage [7] and progesterone [8], which also led to a decrease in early PTBs [7,8] and reduced perinatal deaths [7] or LBW babies [8], respectively. Home uterine monitoring in women at risk of PTB decreased early PTBs and admissions to NICUs [19]. On the other hand, the negative effect of oestrogen supplementation on the primary outcome was also seen in an increase of early PTBs, perinatal deaths and side effects due to treatment [10]. Without altering any other outcome significantly, the administration of any vitamins led to an increase in side effects [13]. No differences in any – primary or secondary – outcome were observed for the remaining seven interventions [9,11,12,14-17].

#### b) Ultrasound screening

None of the reviews [20,21] reported significant group differences for primary (Additional file 3: Table S1b) or secondary outcomes.

#### c) Prevention, detection and management of infection

The review on lower genital tract screening of women without symptoms of lower genital tract infection found a decrease in PTBs <37 weeks compared to no screening [24], whereas metronidazole for the treatment of trichomoniasis led to an increase in PTBs compared to no treatment in asymptomatic women [28] (Additional file 3: Table S1c).With regard to secondary outcomes, lower genital tract screening [24] and antibiotic treatment of asymptomatic bacteriuria [27] lowered the incidence of LBW babies. No effects in primary or secondary outcomes were seen for the remaining four interventions [22,23,25,26].

#### d) Prevention, detection and management of hypertension/pre-eclampsia and management of hyperglycaemia

Antiplatelet agents versus none for primary prevention of pre-eclampsia in women at risk of developing pre-eclampsia [32], routine calcium supplementation in pregnancy [35], and some rest in hospital versus routine activity at home for women with raised blood pressure [39] showed a significant reduction in PTBs <37 weeks (Additional file 3: Table S1d).

The only positive effect in secondary outcomes could be observed for antiplatelet agents in the primary prevention of pre-eclampsia in women at risk of developing pre-eclampsia [32], which decreased the incidence of SGA babies. A negative effect in secondary outcomes – an increase in side-effects – was seen for nitric oxide [30], diuretics [31], antioxidants [34] and any anti-hypertensive drugs [37]. The latter effect could not be observed in the specific analysis of oral beta-blockers for women with mild to moderate hypertension, but in this comparison the intervention group was more likely to give birth to SGA babies [38]. No statistically significant group differences in primary or secondary outcomes were reported for the remaining five interventions [29,33,36,40,41].

#### e) Nutritional supplements and dietary interventions

Significant PTB reductions had been achieved by nutritional advice during pregnancy to increase energy and protein intake [43], zinc [47], as well as magnesium [48] supplementation, whereas vitamin C supplementation led to an increase in PTBs [45] (Additional file 3: Table S1e). An additional effect in secondary outcomes was seen for magnesium supplementation, which also decreased the incidence of LBW and SGA babies [48]. A positive effect solely in secondary outcomes was seen for a balanced protein/energy supplementation (decrease in SGA babies and stillbirths) [43], multiple micronutrient supplementation (decrease of LBW and SGA babies) [50], and marine oil (reduction of early PTBs, but concurrently increasing side effects like belching and an unpleasant taste) [51]. Two reviews reported negative effects in secondary outcomes: High protein supplementation in pregnancy led to an increase in SGA babies [43], and daily iron supplementation [49] elevated side effects. No statistically significant group differences were observed for the remaining four interventions [42,44,46,52].

#### f) Psychosocial interventions and alternative models of care

None of the four interventions [53-56] reported significant changes in PTBs <37 weeks (Additional file 3: Table S1f). The only statistically significant effect in secondary outcomes was seen for the comparison of a reduced number of antenatal care visits with standard antenatal care programmes for women at low risk of developing complications during pregnancy in low- and middle-income settings (increase of miscarriages/perinatal deaths) [55].

#### g) Prevention and management of other morbidities

Interventions to promote smoking cessation led to a significant decrease in PTBs <37 weeks compared to usual care [58]. The treatment of hypothyroidism in pregnancy with levothyroxine versus no treatment also reduced PTBs <37 weeks, while this positive effect could not be observed for selenomethionine [61] (Additional file 3: Table S1g). An additional positive effect in secondary outcomes was only seen for smoking cessation interventions that also decreased LBWs [58]. Heparin in women considered at risk of placental dysfunction reduced the incidence of SGA babies [57] without altering any other outcome. No effect on primary or secondary outcomes was seen for the remaining three interventions [59,60,62].

## Discussion

### Principal findings

This overview of 56 Cochrane systematic reviews primarily analysed the effects of antenatal interventions on PTB before 37 weeks of gestation. Three interventions increased PTB rates significantly, while twelve interventions led to a statistically significant lower incidence of PTBs compared to controls. The remaining interventions failed to demonstrate a significant effect on PTB rates.

### Interventions which increased PTB risk

The three interventions shown in Cochrane reviews to increase PTB incidence were metronidazole treatment in pregnant women with asymptomatic trichomoniasis [28], as well as vitamin C [45] or oestrogen [10] supplementation. Due to various additional negative effects, the latter is no longer in use.

### Interventions which decreased PTB risk

Of the twelve interventions that led to a statistically significant lower incidence of PTBs compared to controls, some observations currently seem to rely on weak evidence due to single, small or poor methodological quality trials (e.g., fetal fibronectin testing [18], bed rest for hypertension during pregnancy [39] and dietary magnesium supplementation[48]). The Cochrane review of antenatal lower genital tract infection screening [24] found a decrease of PTBs and LBW babies (based on a single clinical trial), whereas the previously published review assessing the effect of antibiotics for the treatment of bacterial vaginosis [25] was not able to detect group differences. The remaining eight interventions showed a significant PTB reduction in defined subgroups of pregnant women. The strongest evidence exists for smoking cessation programmes [58] which have been shown to reduce PTB rates and LBW babies, as well as the treatment of clinical hypothyroidism in pregnancy with levothyroxine [61], which is already standard practice. Concerning cerclage, Alfirevic et al. [7] found a statistically significant benefit in PTB reduction compared to no cerclage in women at high risk of pregnancy loss. This effect might be explained by adding seven more recent trials compared to the excluded, older review by Drakeley et al. [63], which had not been able to prove beneficial effects on PTB. The non-significant review result for progesterone application by Meher et al. [29] seems to be contradictory to the significant PTB reduction in Dodd et al. [8]. However, Meher et al. [29] aimed at primarily analysing effects of progesterone administration on pre-eclampsia prevention. It is based on a single study with 168 women from the United States of America who were considered to be at risk of PTB due to active military service. The significant PTB reduction observed by Dodd et al. [8] only applies to women with a previous history of PTB and could not be seen in subgroup analyses for women with multiple pregnancies or other reasons for ‘PTB risk’. Two interventions for women with (high) risk of developing pre-eclampsia reduced PTB risk, but also showed a positive impact on other pregnancy outcomes: These interventions are the administration of low-dose aspirin after 12 weeks of gestation [37] and calcium supplementation [35], though the latter intervention simultaneously led to a small increase in the risk of HELLP syndrome. Based on subgroup analyses in this review, the recommendation for calcium supplementation also applies to women with low dietary calcium intake [35], while no positive effects could be observed in the separate review on calcium supplementation during pregnancy in the general population [42]. Positive effects on PTB in undernourished women or women from low-income countries with high perinatal mortality can be expected for nutritional advice to increase protein and energy intake, balanced energy and protein supplementation [43], as well as zinc supplementation [47].

### Interventions without a significant effect on PTB risk

The remaining antenatal interventions had no (statistically significant) effect on our primary outcome of PTBs < 37 weeks. However, a few interventions showed at least advantages in secondary outcomes: nutritional supplements like fish oil [51] led to a reduction in early PTBs, balanced protein/energy supplementation [43] to fewer SGA babies and stillbirths, and multiple micronutrients [50] to fewer LBW and SGA babies. Drugs, e.g., antibiotic treatment, reduced the number of LBW babies in women with asymptomatic bacteriuria [27], and with heparin treatment, fewer SGA babies were observed in women considered at risk of placental dysfunction [57]. Conversely, some interventions increased the risk of negative effects in secondary outcomes without influencing PTB rates. Increased side effects were observed for daily iron supplementation [49], (any) vitamins in women irrespective of risk of miscarriage [13], (any) antioxidants [34], diuretics [31], as well as for nitric oxide [30] in pre-eclampsia prevention. More SGA babies could be observed when beta-blockers were used in women with mild to moderate hypertension [38], but also for the use of high protein supplementation in pregnancy [43]. Finally, the reduction in the number of antenatal visits [55] led to more miscarriages/ perinatal deaths. About half of the included reviews were not able to detect any statistically significant group difference in primary or secondary outcomes. However, some of these interventions might show positive or negative effects in outcomes not covered in this overview (e.g., additional support during pregnancy reduced the likelihood of caesarean births or antenatal hospital admissions [53]).

### Interventions with unknown influence on PTB risk

As an unintended result of this overview, during the literature selection process we discovered 28 Cochrane reviews which intended to report on PTB < 37, but were not able to find any RCTs reporting PTB < 37 data. These reviews addressed highly relevant and sometimes routinely used interventions like cervical pessary for prevention [64] or risk scoring systems for predicting PTB [65], routine ultrasound in early pregnancy [66], or dietary advice for the prevention of [67] and screening for [68] gestational diabetes. In order to provide some information about potential effects on secondary outcomes considered in this overview, we prepared Table S4 as an additional file ([see Additional file 5]).

### Results against the backdrop of prior research

Compared to the overview of interventions which might influence PTB risk, published in 2008 [5], minimal changes in the conclusions can be observed. The elevated PTB risk of metronidazole was already reported in 2008, and vitamin C supplementation (though in combination with vitamin E for pre-eclampsia prevention) was reported to be ineffective with regard to PTB rates (but not increasing them). Conflicting results of antibiotic and progesterone treatment had already been stated. The beneficial effects of smoking cessation and of low-dose aspirin for women at high risk of developing pre-eclampsia were already known. Regarding cerclage, Iams et al. [5] had already reported beneficial effects only for subgroups of pregnant women (e.g., with a short cervix and a history of PTB). Contrary to the findings of our overview, fetal fibronectin testing (with subsequent metronidazole and erythromycin treatment) was considered to be ineffective, bed rest had not been studied and beneficial effects on PTB rates had neither been reported for calcium supplementation, nor for protein and calorie supplementation. Effects of magnesium or zinc supplementation, oestrogen or levothyroxine treatment had not been mentioned in the article [5] as a primary or secondary preventive measure. The two remaining interventions judged as promising in 2008 [5] did not show a significant effect on our primary outcome, but fish oil led to a reduction in early PTBs in women without pre-eclampsia/IUGR, and antibiotic treatment reduced the number of LBW babies in women with asymptomatic bacteriuria.

In the recently published article by Requejo et al. [69] (based on the WHO *Born Too Soon-Global Action Report on Preterm Birth*[1]), recommendations for antenatal care have been summarised. The authors state that screening and treatment for asymptomatic bacteriuria or bacterial vaginosis ‘may reduce PTBs’, but add that study findings show inconsistent results and therefore urge more research on the relationship between infections and PTB in general. Similarly, they point out that providers might administer nutritional supplements and counselling services, but again refer to the lack of clear beneficial effects shown in clinical trials, especially with regard to the timing of nutritional interventions. They emphasise the need for identifying women at higher risk of PTB, but declare that prospective studies to evaluate risk-screening tools are still needed. According to the authors, this also applies to specialised antenatal clinics for at-risk women, for which evidence of ineffectiveness is based on (older) trials with less comprehensive screening tests. Requejo et al. [69] further confirm the positive findings on progesterone application for defined subgroups of women (singleton pregnancies with short cervix). They also state that small reductions in PTB rates have been reported in Cochrane reviews for treatment of pre-eclampsia (e.g., for calcium supplementation) and that further research is needed for cervical pessary application and cerclage in different subpopulations of women.

Overall, they conclude that ‘clinical trial literature shows a lack of evidence for many of the preventive interventions currently in use’ and explain the paucity of evidence as partly being due to insufficient research on underlying determinants of preterm delivery.

### Strengths and weaknesses of the study

This article is, to our knowledge, the first overview of Cochrane reviews on interventions to reduce PTB rates. The prevention of spontaneous preterm birth appears to be a rapidly evolving maternal and child health topic. 21 of the included reviews were published or updated between 2010 and 2012. Two protocols for reviews directly targeted at PTB prevention (cerclage in multiple pregnancies [70] and the treatment of periodontal disease [71]) were registered in the Cochrane Library at the time of our literature search. Therefore, underlying evidence might have even changed in the time period between the literature search and publication of this overview. Included Cochrane reviews show a wide-ranging publishing period of the underlying primary research. It should be questioned whether results from studies conducted in the 1930s (e.g., on vitamin A supplementation in pregnancy [44]) are transferable to pregnant women in the 21st century at all.

We decided to restrict our overview of reviews solely to Cochrane systematic reviews. Conclusions from this overview therefore only apply to results from Cochrane reviews. We acknowledge that there are other systematic reviews that might be more recent, cover other interventions not targeted by Cochrane reviews so far, or even came to differing conclusions based on different inclusion criteria of primary research.

We prepared this overview mainly in accordance with the corresponding methods chapter of the *Cochrane Handbook of Systematic Reviews of Interventions* (Chapter 22) [6], but we modified, e.g., the presentation of results by deciding to present forest plots for the primary outcome, or by only distinguishing between statistically significant changes – visualised by arrows (Table S3 [see Additional file 4]) – without presenting effect estimations with confidence intervals or other data for our secondary outcomes.

Results of an overview of reviews are influenced by the methodological quality of included systematic reviews. The aggregation of data from different primary studies in systematic reviews may lead to diverging results of the reviews. Similarly, the inclusion of different systematic reviews in an overview of reviews is likely to cause inconsistent results across overviews, depending on the underlying, already aggregated evidence considered. We justified our refraining from a quality assessment of included systematic reviews by referring to quality standards for the preparation of Cochrane reviews defined by the Cochrane collaboration [6]. According to these requirements, Cochrane reviews should fulfil adequate quality standards [72] for the domains judged as important within AMSTAR, a validated tool to assess the methodological quality of systematic reviews [73]. These criteria include an a priori definition of the research question and inclusion criteria, a duplicate study selection and data extraction, a comprehensive literature search including grey literature, the provision of the characteristics of included studies and a list of excluded studies, a quality assessment of included studies and its appropriate consideration in formulating conclusions, the appropriateness of statistical methods used, the assessment of a potential publication bias and, finally, the declaration of potential conflicts of interest. We further decided not to perform a quantitative meta-analysis to estimate a pooled effect size of results because of the obvious heterogeneity of interventions. This predefinition avoided an overestimation of (pooled) effects, given the risk that data from primary studies might have already been included in several reviews. However, we only excluded one Cochrane review [63] that was solely based on RCTs included in a more recent review [7]. Other included systematic reviews might overlap with regard to included RCTs.

In general, an evident drawback of an overview of reviews is the loss of more detailed information by qualitatively aggregating information on a meta-level. For example, women ‘at (high) risk of PTB’ were defined precisely by some Cochrane review authors, while others referred to the trials authors’ definitions, women’s obstetricians judgement or did not specify risk evaluation at all. Drawing general conclusions from the results of an overview is therefore difficult and would be negligent without taking a closer look at individual reviews, included primary research, and contradictory results of subgroup analyses performed.

## Conclusion

The possible effects of a diverse range of interventions on PTB have been evaluated in Cochrane systematic reviews. Few interventions have been demonstrated to be effective and a small number have been found to be harmful. For around half of the interventions evaluated, the Cochrane review concluded that there was insufficient evidence to provide sound recommendations for clinical practice. No RCT evidence is available for a number of potentially relevant interventions.

## Abbreviations

LBW: Low birth weight (less than 2500 grams); NICU: To neonatal intensive care units; PTB: Preterm birth; PTB < 37: Preterm birth at less than 37 weeks of gestation; PTB < 34: Preterm birth at less than 34 weeks of gestation; RCT: Randomised controlled trial; SGA: Small for gestational age (birth weight less than the 10th percentile of gestational age).

## Competing interests

The authors declare that they have no competing interests.

## Authors’ contributions

BP was responsible for the study conception and design. BP and IZ reviewed articles for relevance. BP wrote the first draft of the manuscript. IZ and RW made critical revisions to the manuscript. All authors read and approved the final version.

## Authors’ information

All authors work for the LBI-HTA.

## Supplementary Material

Additional file 1 Search strategy. Click here for file

Additional file 2: Table S2 Characteristics of included reviews [7-62]. Click here for file

Additional file 3: Table S1 Effect estimations for preterm birth prior to 37 weeks of gestation [7-62]. Click here for file

Additional file 4: Table S3 Overview of results for primary and secondary outcomes [7-62]. Click here for file

Additional file 5: Table S4 Overview of Cochrane reviews that intended to report on PTB < 37 weeks, but were not able to identify any RCTs reporting PTB data. Click here for file
