# Supplementary material for: Antenatal interventions to reduce preterm birth: an overview of Cochrane systematic reviews
Source: BMC Res Notes. 2014 Apr 23;7:265. doi: 10.1186/1756-0500-7-265 (PMC4021758; doi:10.1186/1756-0500-7-265)
Supplement: Additional file 1 — Search strategy. [file 1756-0500-7-265-S1.docx]

**Additional file 1: Search strategy**

ID           Search

#1           MeSH descriptor: [Obstetric Labor, Premature] explode all trees and with qualifiers: [Prevention & control - PC]

#2           MeSH descriptor: [Premature Birth] explode all trees and with qualifiers: [Prevention & control - PC]

#3           (premature or preterm or pre term or pre-term) near/3 birth*

#4           (premature or preterm or pre term or pre-term) near/3 deliver*

#5           (preterm or pre term or pre-term) near/3 (labor or labour)

#6           premature near/3 (labor or labour or parturition)

#7           #3 or #4 or #5 or #6

#8           MeSH descriptor: [Preventive Medicine] explode all trees

#9           MeSH descriptor: [Preventive Health Services] explode all trees

#10        preventi*

#11        #8 or #9 or #10

#12        #7 and #11

#13        #1 or #2 or #12

#14        Systematic Review*

#15        #13 and #14
