# Supplementary material for: Antenatal interventions to reduce preterm birth: an overview of Cochrane systematic reviews
Source: BMC Res Notes. 2014 Apr 23;7:265. doi: 10.1186/1756-0500-7-265 (PMC4021758; doi:10.1186/1756-0500-7-265)
Supplement: Additional file 2: Table S2 — Characteristics of included reviews [7-62]. [file 1756-0500-7-265-S2.docx]

**Additional file 2: Table S2: Characteristics of included reviews**

| **2a. Prevention of PTB or miscarriage and detection of PTB risk** | | | | | | |
| --- | --- | --- | --- | --- | --- | --- |
| **First author, year [ref.]** | **Title of Cochrane review** | **Studies published in/ between** | **nStudies** | **nWomen** | **Population** | **PTB outcome** |
|  |  |  |  |  |  |  |
| Alfirevic 2012 [7] | Cervical stitch (cerclage) for preventing preterm birth in singleton pregnancy | 1984-2009 | 12 | 3 328 | Women with singleton pregnancies considered to be at high risk of pregnancy loss | secondary |
| Bamigboye 2003 [10] | Oestrogen supplementation for preventing miscarriages and other adverse pregnancy outcomes | 1950 - 1988 | 7 | 2 839 | All pregnant women | unspecified |
| Dodd 2006 [8] | Prenatal administration of progesterone for preventing preterm birth in women considered to be at risk of preterm birth | 1970 - 2008 | 11 | 2 714 | Pregnant women considered to be at increased risk of PTB | primary |
| Haas 2008 [9] | Progestogen for preventing miscarriage | 1953-2005 | 15 | 2 118 | All pregnant women | secondary |
| Whitworth 2008 [11] | Prophylactic oral betamimetics for preventing preterm labour in singleton pregnancies | 1967 | 1 | 103 | Pregnant women at high risk of preterm delivery with a singleton pregnancy | secondary |
| Yamasmit 2005 [12] | Prophylactic oral betamimetics for reducing preterm birth in women with a twin pregnancy | 1967 - 1990 | 5 | 344 | All pregnant women carrying twins | primary |
| Rumbold 2011 [13] | Vitamin supplementation for preventing miscarriage | 1942 - 2008 | 28 | 96 674 | Pregnant women irrespective of risk of miscarriage | primary |
| Sosa 2004 [15] | Bed rest for singleton pregnancies for preventing preterm birth | 1994 | 1 | 1 266 | Pregnant women at high risk of spontaneous PTB | primary |
| Crowther 2010 [14] | Hospitalisation and bed rest for multiple pregnancy | 1984 - 2005 | 7 | 713 | Women with a multiple pregnancy | primary |
| Alexander 2010 [16] | Repeat digital cervical assessment in pregnancy for identifying women at risk of preterm labour | 1987 - 1994 | 2 | 7 163 | All pregnant women | primary |
| Berghella 2008 [18] | Fetal fibronectin testing for reducing the risk of preterm birth | 2002 - 2007 | 5 | 474 | All pregnant women | primary |
| Berghella 2009 [17] | Cervical assessment by ultrasound for preventing preterm delivery | 1997 - 2007 | 5 | 507 | All pregnant women; main interest in symptomatic women with singleton gestations with signs and/or symptoms of preterm labour; subgroup analyses for asymptomatic women planned | primary |
| Urquhart 2012 [19] | Home uterine monitoring for detecting preterm labour | 1987-1999 | 15 | 6 008 | Pregnant women at risk of PTB | primary |

[ref.] reference number; n number of totally included studies/ women; PTB outcome: Preterm birth was defined as primary, secondary or unspecified outcome in the Cochrane review

| **2b. Ulrasound screening** | | | | | | |
| --- | --- | --- | --- | --- | --- | --- |
| **First author, year [ref.]** | **Title of Cochrane review** | **Studies published in/ between** | **nStudies** | **nWomen** | **Population** | **PTB outcome** |
|  |  |  |  |  |  |  |
| Bricker 2008 [20] | Routine ultrasound in late pregnancy (after 24 weeks’ gestation) | 1984-2003 | 8 | 27 024 | Women in late pregnancy (after 24 weeks’ gestation) in both unselected populations and designated low-risk populations | primary |
| Alfirevic 2010 [21] | Fetal and umbilical Doppler ultrasound in normal pregnancy | 1992-1997 | 5 | 14 185 | Pregnant women in both unselected and low-risk populations | secondary |
| **2c. Prevention, detection and management of infection** | | | | | | |
| **First author, year [ref.]** | **Title of Cochrane review** | **Studies published in/ between** | **nStudies** | **nWomen** | **Population** | **PTB outcome** |
|  |  |  |  |  |  |  |
| Othman 2007 [22] | Probiotics for preventing preterm labour | 1993 - 2010 | 3 | 364 | Pregnant women | primary |
| Thinkamrop 2002 [23] | Prophylactic antibiotic administration during second and third trimester in pregnancy for preventing infectious morbidity and mortality | 1990 - 2006 | 9 | 3 223 | Pregnant women | primary |
| Sangkomkamhang 2008 [24] | Antenatal lower genital tract infection screening and treatment programs for preventing preterm delivery | 2004 | 1 | 4 155 | Pregnant women who are not in labour, have no vaginal bleeding and are without symptoms of lower genital tract infection. | primary |
| McDonald 2007 [25] | Antibiotics for treating bacterial vaginosis in pregnancy | 1991 - 2004 | 15 | 5 888 | Pregnant women with a diagnosis of bacterial vaginosis | unspecified |
| Brocklehurst 1998 [26] | Interventions for treating genital chlamydia trachomatis infection in pregnancy | 1982-1998 | 11 | 1 449 | Women with Chlamydia trachomatis infection | unspecified |
| Gülmezoglu 2011 [28] | Interventions for trichomoniasis in pregnancy | 1983 - 2001 | 2 | 842 | Pregnant women with symptomatic or asymptomatic trichomoniasis | primary |
| Smail 2007 [27] | Antibiotics for asymptomatic bacteriuria in pregnancy | 1960 - 1987 | 14 | 2 636 | Pregnant women found on antenatal screening to have asymptomatic bacteriuria | unspecified |

[ref.] reference number; n number of totally included studies/ women; PTB outcome: Preterm birth was defined as primary, secondary or unspecified outcome in the Cochrane review

| **2d. Prevention, detection and management of hypertension/ pre-eclampsia and hyperglycaemia/ (gestational) diabetes** | | | | | | |
| --- | --- | --- | --- | --- | --- | --- |
| **First author, year [ref.]** | **Title of Cochrane review** | **Studies published in/ between** | **nStudies** | **nWomen** | **Population** | **PTB outcome** |
|  |  |  |  |  |  |  |
|  |  |  |  |  |  |  |
| Meher 2006 [29] | Progesterone for preventing pre-eclampsia and its complications | 1962 - 1983 | 2 | 296 | Pregnant women with normal or high blood pressure without proteinuria | primary |
| Meher 2007 [30] | Nitric-oxide for preventing pre-eclampsia and its complications | 1997 - 2002 | 6 | 310 | All pregnant women | primary |
| Churchill 2007 [31] | Diuretics for preventing pre-eclampsia | 1962 - 1984 | 5 | 1 836 | All pregnant women without pre-eclampsia at trial entry | unspecified |
| Duley 2007 [32] | Antiplatelet agents for preventing pre-eclampsia and its complications | 1985 - 2005 | 59 | 37 560 | Pregnant women considered to be at risk of developing preeclampsia | unspecified |
| Duley 2005 [33] | Altered dietary salt for preventing pre-eclampsia and its complications | 1997 - 1998 | 2 | 603 | Pregnant women with normal or high blood pressure without proteinuria | primary |
| Rumbold 2008 [34] | Antioxidants for preventing pre-eclampsia | 1994 - 2006 | 10 | 6 533 | All pregnant women without established pre-eclampsia | primary |
| Hofmeyr 2011 [35] | Calcium supplementation during pregnancy for preventing hypertensive disorders and related problems | 1987 - 2009 | 13 | 15 730 | Pregnant women, regardless of the risk of hypertensive disorders of pregnancy, but without diagnosed hypertensive disorders | primary |
| Meher 2006 [36] | Exercise or other physical activity for preventing pre-eclampsia and its complications | 1997 - 2000 | 2 | 45 | Pregnant women with normal blood pressure or high blood pressure without proteinuria | primary |
| Abalos 2007 [37] | Antihypertensive drug therapy for mild to moderate hypertension during pregnancy | 1976 - 2002 | 46 | 4 282 | Women with mild to moderate hypertension | unspecified |
| Magee 2003 [38] | Oral beta-blockers for mild to moderate hypertension during pregnancy | 1978-1998 | 29 | 2 501 | Women with mild to moderate hypertension during pregnancy | unspecified |
| Meher 2010 [39] | Bed rest with or without hospitalisation for hypertension during pregnancy | 1982 - 1998 | 4 | 449 | Pregnant women with raised blood pressure | primary |
| Duley 1999 [40] | Plasma volume expansion for treatment of pre-eclampsia | 1980-1993 | 3 | 61 | Women with hypertension during pregnancy, whether or not proteinuria was specified to be present | unspecified |
| Han 2012 [41] | Interventions for pregnant women with hyperglycaemia not meeting gestational diabetes and type 2 diabetes diagnostic criteria | 1989-2011 | 4 | 543 | Pregnant women with hyperglcaemia who do not meet diagnostic criteria for GDM | secondary |

[ref.] reference number; n number of totally included studies/ women; PTB outcome: Preterm birth was defined as primary, secondary or unspecified outcome in the Cochrane review, GDM gestational diabetes mellitus

| **2e. Dietary interventions** |  |  |  |  |  |  |
| --- | --- | --- | --- | --- | --- | --- |
| **First author, year [ref.]** | **Title of Cochrane review** | **Studies published in/ between** | **nStudies** | **nWomen** | **Population** | **PTB outcome** |
|  |  |  |  |  |  |  |
|  |  |  |  |  |  |  |
| Ota 2012 [43] | Antenatal dietary advice and supplementation to increase energy and protein intake | 1973-2009 | 15 | 7 410 | All pregnant women with no systematic illness. | secondary |
| van den Broek 2010 [44] | Vitamin A supplementation during pregnancy for maternal and newborn outcomes | 1931-2010 | 16 | 257 401 | All pregnant women | secondary |
| Rumbold 2005 [45] | Vitamin C supplementation in pregnancy | 1997 - 2003 | 5 | 766 | All pregnant women | primary |
| Rumbold 2005 [46] | Vitamin E supplementation in pregnancy | 1997 - 2002 | 4 | 566 | All pregnant women | primary |
| Mori 2012 [47] | Zinc supplementation for improving pregnancy and infant outcome | 1983-2010 | 20 | 15 001 | All pregnant women with no systemic illness | primary |
| Buppasiri 2011 [42] | Calcium supplementation (other than for preventing or treating hypertension) for improving pregnancy and infant outcomes | 1983-2009 | 21 | 17 212 | All pregnant women | primary |
| Makrides 2001 [48] | Magnesium supplementation in pregnancy | 1988-1997 | 7 | 2 339 | All pregnant women | unspecified |
| Pena-Rosas 2009 [49] | Effects and safety of preventive oral iron or iron + folic acid supplementation for women during pregnancy | 1947 - 2008 | 49 | 23 200 | All pregnant women | primary |
| Duley 1999 [52] | Reduced salt intake compared to normal dietary salt, or high intake, in pregnancy | 1997-1998 | 2 | 603 | Women without pre-eclampsia | unspecified |
| Haider 2006 [50] | Multiple-micronutrient supplementation for women during pregnancy | 1943-2005 | 9 | 15 378 | All pregnant women (except women with HIV) | primary |
| Makrides 2006 [51] | Marine oil and other prostaglandin precursor supplementation for pregnancy uncomplicated by pre-eclampsia or intrauterine growth restriction | 1992 - 2003 | 6 | 2 755 | All pregnant women, regardless of their risk of pre-eclampsia, PTB or IUGR but without established pre-eclampsia or suspected IUGR | unspecified |
| **2f. Psychosocial interventions and alternative models of care** | | | | | | |
| **First author, year [ref.]** | **Title of Cochrane review** | **Studies published in/ between** | **nStudies** | **nWomen** | **Population** | **PTB outcome** |
|  |  |  |  |  |  |  |
| Hodnett 2010 [53] | Support during pregnancy for women at increased risk of low birthweight babies | 1986 - 2001 | 17 | 12 264 | Pregnant women judged to be at risk of having preterm or growth restricted babies | primary |
| Whitworth 2011 [54] | Specialised antenatal clinics for women with a pregnancy at high risk of preterm birth (excluding multiple pregnancy) to improve maternal and infant outcomes | 1989-1994 | 3 | 3 400 | Pregnant women with a singleton pregnancy considered at high risk of preterm labour | primary |
| Dowswell 2010 [55] | Alternative versus standard packages of antenatal care for low-risk pregnancy | 1995-2007 | 7 | 60 724 | Women at low risk of developing complications during pregnancy/ labour | secondary |
| Hatem 2009 [56] | Midwife-led versus other models of care for childbearing women | 1989-2003 | 11 | 12 276 | Pregnant women classified as low and mixed risk of complications | unspecified |

[ref.] reference number; n number of totally included studies/ women; PTB outcome: Preterm birth was defined as primary, secondary or unspecified outcome in the Cochrane review; HIV Human Immunodeficiency Virus; IUGR intrauterine growth restriction

| **2g. Prevention and management of other morbidities** | | | | | | |
| --- | --- | --- | --- | --- | --- | --- |
| **First author, year [ref.]** | **Title of Cochrane review** | **Studies published in/ between** | **nStudies** | **nWomen** | **Population** | **PTB outcome** |
|  |  |  |  |  |  |  |
| Kramer 2010 [59] | Aerobic exercise for women during pregnancy | 1976 - 2006 | 14 | 1 014 | Healthy pregnant women | unspecified |
| Muktabhant 2012 [60] | Interventions for preventing excessive weight gain during pregnancy | 1968-2011 | 28 | 3 976 | Pregnant women | secondary |
| Lumley 2009 [58] | Interventions for promoting smoking cessation during pregnancy | 1975 - 2008 | 72 | 25 001 | Pregnant women | unspecified |
| Dodd 2010 [57] | Antithrombotic therapy for improving maternal or infant health outcomes in women considered at risk of placental dysfunction | 1995 - 2009 | 5 | 484 | Pregnant women considered at risk of placental dysfunction | primary |
| Reid 2010 [61] | Interventions for clinical and subclinical hyopthyroidism in pregnancy | 2004 - 2007 | 3 | 314 | Pregnant women with a diagnosis of hypothyroidism, subclinical hypothyroidism or isolated maternal hypothyroxinaemia. | primary |
| Say 1996 [62] | Calcium channel blockers for potential impaired fetal growth | 1985 | 1 | 100 | Women either at high risk or with suspected impaired fetal growth | unspecified |

[ref.] reference number; n number of totally included studies/ women; PTB outcome: Preterm birth was defined as primary, secondary or unspecified outcome in the Cochrane review
