# Supplementary material for: Antenatal interventions to reduce preterm birth: an overview of Cochrane systematic reviews
Source: BMC Res Notes. 2014 Apr 23;7:265. doi: 10.1186/1756-0500-7-265 (PMC4021758; doi:10.1186/1756-0500-7-265)
Supplement: Additional file 3: Table S1 — Effect estimations for preterm birth prior to 37 weeks of gestation [7-62]. [file 1756-0500-7-265-S3.docx]

**Additional file 3: Table S1: Effect estimations for preterm birth prior to 37 weeks of gestation**

**1a. Prevention of PTB or miscarriage and detection of PTB risk**

| **Author, year** [**ref.**] | **Intervention** | **Population**  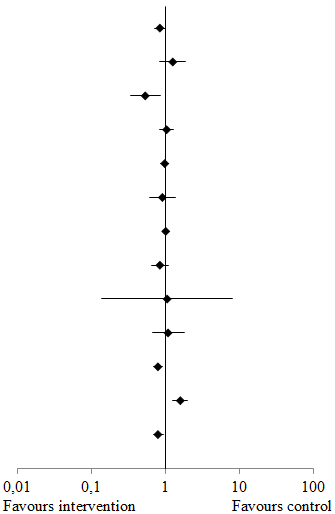 | **Effect size, 95% CI** | **n RCTs** | **n Women** |
| --- | --- | --- | --- | --- | --- |
| Urquhart et al, 2012 [19] | Home uterine monitoring | At risk of PTB |  | 8 | 4 834 |
| Berghella et al, 2009 [17] | TVU knowledge | Twin pregnancy |  | 1 | 125 |
| Berghella et al, 2008 [18] | FFT knowledge | All women |  | 3 | 275 |
| Alexander et al, 2010 [16] | Digital cervical examination | All women |  | 2 | 6 070 |
| Crowther et al, 2010 [14] | Bed rest in hospital | Multiple pregnancy |  | 7 | 713 |
| Sosa et al, 2004 [15] | Bed rest | At high risk of PTB |  | 1 | 1 266 |
| Rumbold et al, 2011 [13] | Any vitamins | All women |  | 8 | 27 414 |
| Yamasmit et al, 2005 [12] | Betamimetics | Twin pregnancy |  | 4 | 276 |
| Whitworth et al, 2008 [11] | Betamimetics | At high risk of PTB |  | 1 | 64 |
| Haas et al, 2008 [9] | Progestogen | All women |  | 7 | 946 |
| Dodd et al, 2006 [8] | Progesterone | With pervious PTB |  | 4 | 1 255 |
| Bamigboye et al, 2003 [10]* | Diethylstilbestrol | All women |  | 3 | 2 173 |
| Alfirevic et al, 2012 [7] | Cerclage | At high risk of pregnancy loss |  | 9 | 2 898 |
|  |  |  |  |  |  |

**1b. Ultrasound screening**

| **Author, year** [**ref.** ] | **Intervention** | 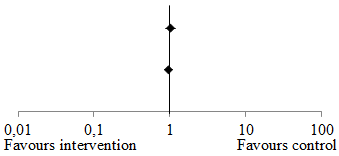**Population** | **Effect size, 95% CI** | **n RCTs** | **n Women** |
| --- | --- | --- | --- | --- | --- |
| Alfirevic et al, 2010 [21] | Routine Doppler ultrasound | All women |  | **4** | 12 162 |
| Bricker et al, 2008 [20] | Routine ultrasound>24 weeks | All women |  | **2** | 17 151 |

**1c. Prevention, detection and management of infection**

| **Author, year** [**ref.**] | **Intervention** | **Population** | **Effect size, 95% CI** | **n RCTs** | **n Women** |
| --- | --- | --- | --- | --- | --- |
| Smail et al, 2007 [27]* | Antibiotics | 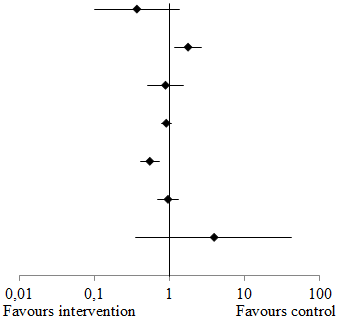With asymptomatic bacteriuria |  | 3 | 412 |
| Gülmezoglu et al, 2011 [28] | Metronidazole | With asymptomatic trichomoniasis |  | 1 | 604 |
| Brocklehurst et al, 1998 [26] | Antibiotics | With chlamydia trachomatis infection |  | 1 | 405 |
| McDonald et al, 2007 [25] | Antibiotics | With bacterial vaginosis |  | 12 | 5 888 |
| Sangkomkamhang et al, 2008 [24] | Lower genital tract screening | All women |  | 1 | 4 155 |
| Thinkamrop et al, 2002 [23] | Prophylactic antibiotics | All women |  | 6 | 1 416 |
| Othman et al, 2007 [22] | Probiotics | All women |  | 1 | 238 |
|  |  |  |  |  |  |

**1d. Prevention, detection and management of hypertension/ pre-eclampsia and hyperglycaemia/ (gestational) diabetes**

| **Author, year** [**ref.** ] | **Intervention** | **Population**  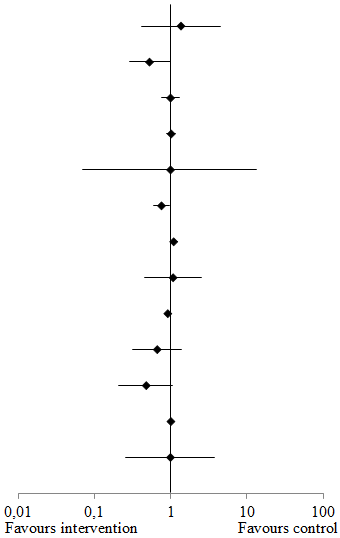 | **Effect size, 95% CI** | **n RCTs** | **n Women** |
| --- | --- | --- | --- | --- | --- |
| Duley et al, 1999 [40] | Plasma volume expansion | With hypertension |  | 1 | 32 |
| Meher et al, 2010 [39] | Somerest in hospital | With raised blood pressure |  | 1 | 218 |
| Magee et al, 2003 [38] | Beta-blockers | With mild/ moderate hypertension |  | 8 | 962 |
| Abalos et al, 2007 [37] | Antihypertensive drugs | With mild/ moderate hypertension |  | 14 | 1 992 |
| Meher et al, 2006 [36] | Regular aerobic exercise | Without proteinuria |  | 2 | 45 |
| Hofmeyr et al, 2011 [35] | Calcium | Without hypertension |  | 11 | 15 275 |
| Rumbold et al,2008 [34] | Antioxidants | Without eclampsia |  | 5 | 5 198 |
| Duley et al, 2005 [33] | Low salt intake | Without proteinuria |  | 1 | 242 |
| Duley et al, 2007 [32] | Antiplatelets | At risk of pre-eclampsia |  | 29 | 31 151 |
| Churchill et al, 2007 [31] | Diuretics | Without pre-eclampsia |  | 2 | 465 |
| Meher et al, 2007 [30] | Nitric oxide | All women |  | 3 | 154 |
| Meher et al, 2006 [29] | Progesterone | Without proteinuria |  | 3 | 1 313 |
| Han et al, 2012 [41] | Intensive management | With hyperglcaemia, not GDM |  | 2 | 138 |
|  |  |  |  |  |  |

**1e. Nutritional supplements and dietary interventions**

| **Author, year** [**ref.**] | **Intervention** | **Population** | **Effect size, 95% CI** | **n RCTs** | **n Women** |
| --- | --- | --- | --- | --- | --- |
| Makrides et al, 2006 [51] | Fishoil | 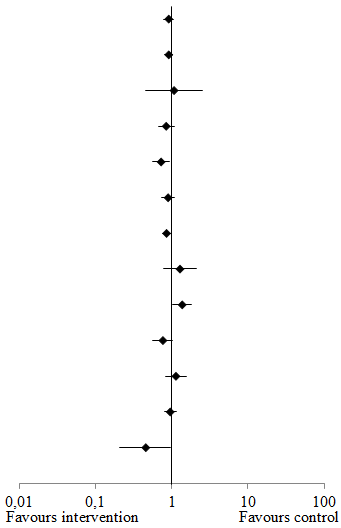Without pre-eclampsia or IUGR |  | 5 | 1 916 |
| Haider et al, 2006 [50] | Multiple micronutrients | Without HIV |  | 6 | 5 756 |
| Duley et al, 1999 [52] | Low salt intake | Without pre-eclampsia |  | 1 | 242 |
| Pena-Rosas et al, 2009 [49] | Daily iron | All women |  | 8 | 5 730 |
| Makrides et al, 2001 [48] | Magnesium | All women |  | 5 | 2 275 |
| Buppasiri et al, 2011 [42] | Calcium | All women |  | 12 | 15 615 |
| Mori et al, 2012 [47] | Zinc | Without systematic illness |  | 16 | 7 637 |
| Rumbold et al, 2005 [46] | Vitamin E | All women |  | 2 | 383 |
| Rumbold et al, 2005 [45] | Vitamin C | All women |  | 3 | 583 |
| van den Broek et al, 2010 [44] | Vitamin A | All women |  | 4 | 1 937 |
| Ota et al, 2012 [43] | High protein supplementation | Without systematic illness |  | 1 | 505 |
| Ota et al, 2012 [43] | Balanced protein/ energy supplementation | Without systematic illness |  | 5 | 3 384 |
| Ota et al, 2012 [43] | Nutritional advice | Without systematic illness |  | 2 | 449 |
|  |  |  |  |  |  |

**1f. Psychosocial interventions and alternative models of care**

| **Author, year** [**ref.**] | **Intervention** | **Population** | **Effect size, 95% CI** | **n RCTs** | **n Women** |
| --- | --- | --- | --- | --- | --- |
| Hatem et al, 2009 [56] | Midwife-led models of care | 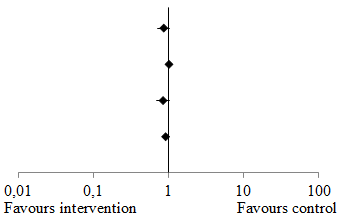At low and mixed risk of complications |  | 5 | 7 516 |
| Dowswell et al, 2010 [55] | Reduced number of antenatal care visits | At low risk of complications |  | 7 | 60 724 |
| Whitworth et al, 2011 [54] | Specialised antenatal care | Singleton pregnancies at high risk of PTB |  | 3 | 3 400 |
| Hodnett et al, 2010 [53] | Additional support | At risk of PTB or IUGR |  | 11 | 10 429 |
|  |  |  |  |  |  |

**1g. Prevention and management of other morbidities**

| **Author, year** [**ref.**] | **Intervention** | 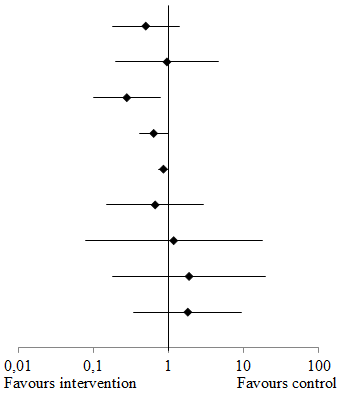**Population** | **Effect size, 95% CI** | **n RCTs** | **n Women** |
| --- | --- | --- | --- | --- | --- |
| Say et al, 1996 [62] | Flunarizine | At high risk/ suspected IUGR |  | 1 | 100 |
| Reid et al, 2010 [61] | Selenomethionine | With (subclinical) hypothyroidism |  | 1 | 151 |
| Reid et al, 2010 [61] | Levothyroxine | With (subclinical) hypothyroidism |  | 1 | 105 |
| Dodd et al, 2010 [57] | Heparin alone or with other medication | At risk of placental dysfunction |  | 3 | 237 |
| Lumley et al, 2009 [58]^+^ | Smoking cessation interventions | All (smoking) women |  | 14 | 11 930 |
| Muktabhant et al, 2012 [60] | Regular weight measurement | All women |  | 1 | 235 |
| Kramer et al, 2010 [59] | Reduction in exercise | Physically fit, healthy |  | 1 | 61 |
| Kramer et al, 2010 [59] | Increase in exercise | Overweight, healthy |  | 1 | 72 |
| Kramer et al, 2010 [59] | Increase in exercise | Sedentary, healthy |  | 3 | 111 |
|  |  |  |  |  |  |

All interventions are compared with no intervention, placebo or routine care (for more details on population and interventions see Additional File 2); effect sizes (risk or odds ratios) with the 95% confidence interval (CI) are presented as forest plots (for more details on statistical methods used see Additional File 3); interventions that showed statistically significant group differences in PTBs <37 weeks are highlighted (green: decrease in PTBs; red: increase in PTBs)

* preterm birth data for PTB less than 38 weeks of gestation

**^+^** preterm birth data for PTB less than 37 or 36 weeks of gestation

Abbreviations: ref. reference number; n number of; PTB preterm birth; TVU transvaginal ultrasound; FFT fetal fibronectin testing; GDM gestational diabetes mellitus; IUGR intra uterine growth restriction; HIV human immunodeficiency virus
