# Supplementary material for: Antenatal interventions to reduce preterm birth: an overview of Cochrane systematic reviews
Source: BMC Res Notes. 2014 Apr 23;7:265. doi: 10.1186/1756-0500-7-265 (PMC4021758; doi:10.1186/1756-0500-7-265)
Supplement: Additional file 4: Table S3 — Overview of results for primary and secondary outcomes [7-62]. [file 1756-0500-7-265-S4.docx]

**Additional file 4: Table S3: Overview of results for primary and secondary outcomes**

| **3a. Prevention of PTB or miscarriage and detection of PTB risk** | | | | | | | | | | | | | | |
| --- | --- | --- | --- | --- | --- | --- | --- | --- | --- | --- | --- | --- | --- | --- |
|  |  | **Primary outcome** | | | | |  | **Secondary outcomes** | | | | | | |
| **First author, year [ref.]** | **Comparison (population)** | **nT** | **nW** | **Statistical method** | **Effect size** | **PTB <37** |  | **PTB <32-34** | **LBW** | **SGA** | **NICU** | **Stillb.** | **Miscar./ p.death** | **Side effects** |
| Alfirevic 2012 [7] | Cerclage vs no cerclage (women with singleton pregnancies considered to be at high risk of pregnancy loss) | 9 | 2 898 | Risk Ratio (M-H, Random, 95% CI) | **0.8 [0.69, 0.95]** | **🡫** |  | **🡫** | NR | NR | NR | 🡨🡪 | 🡨🡪 | 🡨🡪 |
| Bamigboye 2003 [10] | Diethylstilbestrol versus control (all women) | 3 | 2 173 | Risk Ratio (M-H, Fixed, 95% CI) | **1.61 [1.28, 2.02]** | **🡩** |  | NR | **🡩** | NR | NR | 🡨🡪 | **🡩** | **🡩** |
| Dodd 2006 [8] | Progesterone vs. placebo (women with previous history of spontaneous PTB) | 4 | 1 255 | Risk Ratio (M-H, Fixed, 95% CI) | **0,8 [0.7, 0.92]** | **🡫** |  | **🡫** | **🡫** | NR | NR | NR | 🡨🡪 | NR |
| Haas 2008 [9] | Progestogen versus placebo/no treatment (all women) | 7 | 946 | Peto Odds Ratio (Peto, Fixed, 95% CI) | 1.1 [0.67, 1.81] | 🡨🡪 |  | NR | NR | NR | NR | NR | 🡨🡪 | NR |
| Whitworth 2008 [11] | Betamimetic versus placebo (women at high risk of preterm delivery with a singleton pregnancy) | 1 | 64 | Odds Ratio (M-H, Fixed, 95% CI) | 1.07 [0.14, 8.09] | 🡨🡪 |  | NR | 🡨🡪 | NR | NR | NR | 🡨🡪 | 🡨🡪 |
| Yamasmit 2005 [12] | Oral betamimetic versus placebo (all women carrying twins) | 4 | 276 | Risk Ratio (M-H, Fixed, 95% CI) | 0.85 [0.65, 1.1] | 🡨🡪 |  | 🡨🡪 | 🡨🡪 | 🡨🡪 | NR | NR | NR | NR |
| Rumbold 2011 [13] | Any vitamins vs. no vitamins or minimal vitamins (women irrespective of risk of miscarriage) | 8 | 27 414 | Relative Risk (Fixed, 95% CI) | 1.02 [0.94, 1.1] | 🡨🡪 |  | 🡨🡪 | NR | 🡨🡪 | 🡨🡪 | 🡨🡪 | 🡨🡪 | **🡩** |
| Sosa 2004 [15] | Bed rest vs. no intervention (women at high risk of spontaneous PTB) | 1 | 1 266 | Risk Ratio (M-H, Fixed, 95% CI) | 0.92 [0.62, 1.37] | 🡨🡪 |  | NR | NR | NR | NR | NR | NR | NR |
| Crowther 2010 [14] | Hospitalisation for bed rest vs. selective admission if complications (women with a multiple pregnancy) | 7 | 713 | Risk Ratio (IV, Random, 95% CI) | 0.99 [0.86, 1.13] | 🡨🡪 |  | 🡨🡪 | 🡨🡪 | NR | 🡨🡪 | 🡨🡪 | 🡨🡪 | NR |
| Alexander 2010 [16] | Systematic digital cervical examination versus no examination unless medically indicated (all women) | 2 | 6 070 | Odds Ratio (M-H, Fixed, 95% CI) | 1.05 [0.85, 1.31] | 🡨🡪 |  | 🡨🡪 | 🡨🡪 | NR | 🡨🡪 | 🡨🡪 | NR | NR |
| Berghella 2008 [18] | Fetal fibronectin knowledge versus no knowledge (all women) | 3 | 275 | Risk Ratio (M-H, Fixed, 95% CI) | **0.54 [0.34, 0.87]** | **🡫** |  | 🡨🡪 | 🡨🡪 | NR | NR | NR | NR | NE |
| Berghella 2009 [17] | TVU knowledge vs. no knowledge (all women without PTL and twin pregancy) | 1 | 125 | Risk Ratio (M-H, Fixed, 95% CI) | 1.27 [0.85, 1.9] | 🡨🡪 |  | 🡨🡪 | NR | NR | NR | NR | NR | NR |
| Urquhart 2012 [19] | Home uterine monitoring versus standard care (women at risk of PTB) | 8 | 4 834 | Risk Ratio (M-H, Random, 95% CI) | 0.85 [0.72, 1.01] | 🡨🡪 |  | **🡫** | NR | NR | **🡫** | NR | NR | NR |

Group differences: **🡩**statistically significant increase; **🡫**statistically significant reduction; 🡨🡪no statistically significant group difference, NR not reported

[ref.] reference number; nT number of included RCTs; nW number of included women, PTB Preterm birth less than 37 or 32-34 weeks of gestation, LBW low birthweight, SGA small for gestational age, NICU neonatal intensive care unit (admission); Stillb. stillbirth, Miscar. Miscarriage; p.death perinatal death; TVU trans vaginal ultrasound; PTL preterm labour

| **3b. Ultrasound screening** | | | | | | | | | | | | | | |
| --- | --- | --- | --- | --- | --- | --- | --- | --- | --- | --- | --- | --- | --- | --- |
|  |  | **Primary outcome** | | | | |  | **Secondary outcomes** | | | | | | |
| **First author, year [ref.]** | **Comparison (population)** | **nT** | **nW** | **Statistical method** | **Effect size** | **PTB <37** |  | **PTB <32-34** | **LBW** | **SGA** | **NICU** | **Stillb.** | **Miscar./ p.death** | **Side effects** |
| Bricker 2008 [20] | Routine ultrasound > 24 weeks vs no/concealed/selective ultrasound > 24 weeks (in unselected and designated low-risk populations) | 2 | 17 151 | Risk Ratio (M-H, Fixed, 95% CI) | 0.96 [0.85, 1.08] | 🡨🡪 |  | NR | 🡨🡪 | 🡨🡪 | 🡨🡪 | 🡨🡪 | 🡨🡪 | NR |
| Alfirevic 2010 [21] | All routine Doppler ultrasound versus no Doppler ultrasound (in unselected and designated low-risk populations) | 4 | 12 162 | Risk Ratio (M-H, Fixed, 95% CI) | 1.02 [0.87, 1.18] | 🡨🡪 |  | NR | NR | NR | 🡨🡪 | 🡨🡪 | 🡨🡪 | NR |
| **3c. Prevention, detection and management of infection** | | | | | | | | | | | | | | |
|  |  | **Primary outcome** | | | | |  | **Secondary outcomes** | | | | | | |
| **First author, year [ref.]** | **Comparison (population)** | **nT** | **nW** | **Statistical method** | **Effect size** | **PTB <37** |  | **PTB <32-34** | **LBW** | **SGA** | **NICU** | **Stillb.** | **Miscar./ p.death** | **Side effects** |
| Othman 2007 [22] | Any probiotics versus any type of control (all women) | 1 | 238 | Risk Ratio (M-H, Fixed, 95% CI) | 3.95 [0.36, 42.91] | 🡨🡪 |  | 🡨🡪 | NR | NR | NR | NR | NR | NR |
| Thinkhamrop 2002 [23] | Prophylactic antibiotics versus placebo (all women irrespective of risk of PTB) | 6 | 1 416 | Risk Ratio (M-H, Random, 95% CI) | 0.96 [0.7, 1.33] | 🡨🡪 |  | NR | 🡨🡪 | 🡨🡪 | 🡨🡪 | NR | 🡨🡪 | NE |
| Sangkomkamhang 2008 [24] | Lower genital tract infection screening versus no screening (women who are not in labour, have no vaginal bleeding and are without symptoms of lower genital tract infection) | 1 | 4 155 | Risk Ratio (M-H, Fixed, 95% CI) | **0.55 [0.41, 0.75]** | **🡫** |  | NR | **🡫** | NR | NE | NR | NR | NE |
| McDonald 2007 [25] | Any antibiotic versus placebo/no treatment (all women with a diagnosis of bacterial vaginosis) | 12 | 5 888 | Peto Odds Ratio (Peto, Fixed, 95% CI | 0.91 [0.78, 1.06] | 🡨🡪 |  | 🡨🡪 | 🡨🡪 | NR | 🡨🡪 | NR | 🡨🡪 | 🡨🡪 |
| Brocklehurst 1998 [26] | Antibiotic therapy versus placebo or no therapy (women with Chlamydia trachomatis infection) | 1 | 405 | Peto Odds Ratio (Peto, Fixed, 95% CI) | 0.89 [0.51, 1.56] | 🡨🡪 |  | NR | NR | NR | NR | NR | NR | 🡨🡪 |
| Gülmezoglu 2011 [28] | Metronidazole versus no treatment (women with asymptomatic trichomoniasis) | 1 | 604 | Risk Ratio (M-H, Fixed, 95% CI) | **1.78 [1.19, 2.66]** | **🡩** |  | NR | 🡨🡪 | NR | NR | NR | NR | NR |
| Smaill 2007 [27] | (Any) antibiotic versus no treatment (women with asymptomatic bacteriuria) | 3 | 412 | Odds Ratio (M-H, Random, 95% CI) | 0.37 [0.1, 1.36] | 🡨🡪 |  | NR | **🡫** | NR | NR | NR | NR | NR |

Group differences: **🡩**statistically significant increase; **🡫**statistically significant reduction; 🡨🡪no statistically significant group difference, NR not reported

[ref.] reference number; nT number of included RCTs; nW number of included women, PTB Preterm birth less than 37 or 32-34 weeks of gestation, LBW low birthweight, SGA small for gestational age, NICU neonatal intensive care unit (admission); Stillb. stillbirth, Miscar. Miscarriage; p.death perinatal death

| **3d. Prevention, detection and management of hypertension/ pre-eclampsia and hyperglycaemia/ (gestational) diabetes** | | | | | | | | | | | | | | |
| --- | --- | --- | --- | --- | --- | --- | --- | --- | --- | --- | --- | --- | --- | --- |
|  |  | **Primary outcome** | | | | |  | **Secondary outcomes** | | | | | | |
| **First author, year [ref.]** | **Comparison (population)** | **nT** | **nW** | **Statistical method** | **Effect size** | **PTB <37** |  | **PTB <32-34** | **LBW** | **SGA** | **NICU** | **Stillb.** | **Miscar./ p.death** | **Side effects** |
| Meher 2006 [29] | Progesterone versus placebo/no treatment ( women with normal or high blood pressure without proteinuria) | 3 | 1 313 | Risk Ratio (M-H, Fixed, 95% CI) | 1.01 [0.93, 1.1] | 🡨🡪 |  | NR | NR | 🡨🡪 | NR | 🡨🡪 | NR | NE |
| Meher 2007 [30] | Nitric oxide (all drugs) versus placebo/no intervention (all women) | 3 | 154 | Risk Ratio (M-H, Fixed, 95% CI) | 0.48 [0.21, 1.07] | 🡨🡪 |  | NR | NR | 🡨🡪 | 🡨🡪 | NR | 🡨🡪 | **🡩** |
| Churchill 2007 [31] | Diuretic versus placebo or no treatment (women without pre-eclampsia at trial entry) | 2 | 465 | Risk Ratio (M-H, Fixed, 95% CI) | 0.67 [0.32, 1.41] | 🡨🡪 |  | NR | NR | NE | NR | 🡨🡪 | 🡨🡪 | **🡩** |
| Duley 2007 [32] | Antiplatelet agents versus placebo/no antiplatelet for primary prevention (women considered to be at risk of developing preeclampsia) | 29 | 31 151 | Risk Ratio (M-H, Fixed, 95% CI) | **0.92 [0.88, 0.97]** | **🡫** |  | NR | 🡨🡪 | **🡫** | 🡨🡪 | 🡨🡪 | 🡨🡪 | NR |
| Duley 2005 [33] | Low vs. normal salt intake (women with normal or high blood pressure without proteinuria) | 1 | 242 | Risk Ratio (M-H, Fixed, 95% CI) | 1.08 [0.46, 2.56] | 🡨🡪 |  | NR | NR | 🡨🡪 | 🡨🡪 | NR | 🡨🡪 | NR |
| Rumbold 2008 [34] | Any antioxidants versus control or placebo (women without established pre-eclampsia) | 5 | 5 198 | Risk Ratio (M-H, Fixed, 95% CI) | 1.1 [0.99, 1.22] | 🡨🡪 |  | 🡨🡪 | NR | 🡨🡪 | 🡨🡪 | 🡨🡪 | 🡨🡪 | **🡩** |
| Hofmeyr 2011 [35] | Routine calcium supplementation in pregnancy (women, regardless of the risk of hypertensive disorders of pregnancy, but without diagnosed hypertensive disorders) | 11 | 15 275 | Risk Ratio (M-H, Random, 95% CI) | **0.76 [0.6, 0.97]** | **🡫** |  | NR | 🡨🡪 | 🡨🡪 | 🡨🡪 | 🡨🡪 | NR | NR |
| Meher 2006 [36] | Regular aerobic exercise versus normal physical activity (women with normal blood pressure or high blood pressure without proteinuria) | 2 | 45 | Risk Ratio (M-H, Fixed, 95% CI) | 1.0 [0.07, 13.3] | 🡨🡪 |  | NR | NR | 🡨🡪 | NR | NR | NR | NR |
| Abalos 2007 [37] | Any antihypertensive drug versus none (women with mild to moderate hypertension) | 14 | 1 992 | Risk Ratio (M-H, Fixed, 95% CI) | 1.02 [0.89, 1.16] | 🡨🡪 |  | 🡨🡪 | NR | 🡨🡪 | 🡨🡪 | 🡨🡪 | 🡫/🡨🡪 | **🡩** |
| Magee 2003 [38] | Beta-blocker versus placebo/no beta-blocker (women with mild to moderate hypertension during pregnancy) | 8 | 962 | Risk Ratio (M-H, Fixed, 95% CI) | 1.0 [0.76, 1.3] | 🡨🡪 |  | NR | NR | **🡩** | 🡨🡪 | NR | 🡨🡪 | 🡨🡪 |
| Meher 2010 [39] | Some rest in hospital versus routine activity at home (women with raised blood pressure) | 1 | 218 | Risk Ratio (M-H, Fixed, 95% CI) | **0.53 [0.29, 0.99]** | **🡫** |  | 🡨🡪 | NR | 🡨🡪 | 🡨🡪 | 🡨🡪 | 🡨🡪 | NR |
| Duley 1999 [40] | Plasma volume expansion versus no expansion (women with hyper-tension, whether or not proteinuria was specified to be present) | 1 | 32 | Risk Ratio (M-H, Fixed, 95% CI) | 1.37 [0.42, 4.51] | 🡨🡪 |  | NR | 🡨🡪 | NR | NR | NR | 🡨🡪 | NR |
| Han 2012 [41] | Intensive management versus routine care (pregnant women with hyperglcaemia who do not meet diagnostic criteria for GDM) | 2 | 138 | Risk Ratio (M-H, Fixed, 95% CI) | 1.0 [0.26, 3.82] | 🡨🡪 |  | NR | NR | 🡨🡪 | 🡨🡪 | NR | NR | NR |

Group differences: **🡩**statistically significant increase; **🡫**statistically significant reduction; 🡨🡪no statistically significant group difference, NR not reported

[ref.] reference number; nT number of included RCTs; nW number of included women, PTB Preterm birth less than 37 or 32-34 weeks of gestation, LBW low birthweight, SGA small for gestational age, NICU neonatal intensive care unit (admission); Stillb. stillbirth, Miscar. Miscarriage; p.death perinatal death; GDM gestational diabetes mellitus

| **3e. Nutritional supplements and dietary interventions** | | | | | | | | | | | | | | |
| --- | --- | --- | --- | --- | --- | --- | --- | --- | --- | --- | --- | --- | --- | --- |
|  |  | **Primary outcome** | | | | |  | **Secondary outcomes** | | | | | | |
| **First author, year [ref.]** | **Comparison (population)** | **nT** | **nW** | **Statistical method** | **Effect size** | **PTB <37** |  | **PTB <32-34** | **LBW** | **SGA** | **NICU** | **Stillb.** | **Miscar./ p.death** | **Side effects** |
| Ota 2012 [43] | Nutritional advice during pregnancy (women with no systematic illness) | 2 | 449 | Risk Ratio (M-H, Fixed, 95% CI) | **0.46 [0.21, 0.98]** | **🡫** |  | NR | NR | 🡨🡪 | NR | 🡨🡪 | NR | NR |
| Ota 2012 [43] | Balanced protein/ energy supplementation in pregnancy (women with no systematic illness) | 5 | 3 384 | Risk Ratio (M-H, Fixed, 95% CI) | 0.96 [0.8, 1.16] | 🡨🡪 |  | NR | NR | **🡫** | NR | **🡫** | NR | NR |
| Ota 2012 [43] | High protein supplementation in pregnancy (women with no systematic illness) | 1 | 505 | Risk Ratio (M-H, Fixed, 95% CI) | 1.14 [0.83, 1.56] | 🡨🡪 |  | NR | NR | **🡩** | NR | 🡨🡪 | NR | NR |
| van den Broek 2010 [44] | Vitamin A alone versus placebo or no treatment (all women) | 4 | 1 937 | Risk Ratio (M-H, Fixed, 95% CI) | 0.77 [0.57, 1.04] | 🡨🡪 |  | NR | 🡨🡪 | NR | NR | 🡨🡪 | 🡨🡪 | NR |
| Rumbold 2005 [45] | Vitamin C supplementation alone or in combination with other supplements compared with placebo (all women) | 3 | 583 | Risk Ratio (M-H, Fixed, 95% CI) | **1.38 [1.04, 1.82]** | **🡩** |  | NR | NR | NR | 🡨🡪 | 🡨🡪 | 🡨🡪 | 🡨🡪 |
| Rumbold 2005 [46] | Any vitamin E supplementation compared with placebo (all women) | 2 | 383 | Risk Ratio (M-H, Fixed, 95% CI) | 1.29 [0.78, 2.15] | 🡨🡪 |  | NR | NR | NR | 🡨🡪 | 🡨🡪 | 🡨🡪 | 🡨🡪 |
| Mori 2012 [47] | Zinc supplementation versus no zinc with or without placebo (women with no systemic illness) | 16 | 7 637 | Risk Ratio (M-H, Fixed, 95% CI) | **0.86 [0.76, 0.97]** | **🡫** |  | NR | 🡨🡪 | 🡨🡪 | NR | 🡨🡪 | NR | NR |
| Buppasiri 2011 [42] | Calcium supplementation versus placebo or no treatment (all women) | 12 | 15 615 | Ratio (M-H, Random, 95% CI) | 0.9 [0.73, 1.11] | 🡨🡪 |  | 🡨🡪 | 🡨🡪 | NR | 🡨🡪 | 🡨🡪 | 🡨🡪 | 🡨🡪 |
| Makrides 2001 [48] | Magnesium vs. control (all women) | 5 | 2 275 | Risk Ratio (M-H, Fixed, 95% CI) | **0.73 [0.57, 0.94]** | **🡫** |  | NR | **🡫** | **🡫** | 🡨🡪 | 🡨🡪 | 🡨🡪 | 🡨🡪 |
| Pena-Rosas 2009 [49] | Daily iron alone versus no intervention/placebo (all women) | 8 | 5 730 | Risk Ratio (M-H, Random, 95% CI) | 0.85 [0.67, 1.09] | 🡨🡪 |  | 🡨🡪 | 🡨🡪 | 🡨🡪 | 🡨🡪 | NR | 🡨🡪 | **🡩** |
| Duley 1999 [52] | Low vs. normal salt intake in pregnancy (women without pre-eclampsia) | 1 | 242 | Risk Ratio (M-H, Fixed, 95% CI) | 1.08 [0.46, 2.56] | 🡨🡪 |  | NR | 🡨🡪 | 🡨🡪 | NR | NR | 🡨🡪 | NR |
| Haider 2006 [50] | Multiple micronutrients versus controls; no supplements, placebo or less than two micronutrients (all women except women with HIV) | 6 | 5 756 | Risk Ratio (M-H, Fixed, 95% CI) | 0.92 [0.82, 1.04] | 🡨🡪 |  | NR | **🡫** | **🡫** | NR | NR | 🡨🡪 | NR |
| Makrides 2006 [51] | Prostagladin precursor supplementation (all data only for fish oil) versus none or placebo (all women, regardless of their risk of pre-eclampsia, PTB or IUGR but without established pre-eclampsia or suspected IUGR) | 5 | 1 916 | Risk Ratio (M-H, Fixed, 95% CI) | 0.92 [0.79, 1.07] | 🡨🡪 |  | **🡫** | 🡨🡪 | 🡨🡪 | 🡨🡪 | 🡨🡪 | 🡨🡪 | **🡩** |

Group differences: **🡩**statistically significant increase; **🡫**statistically significant reduction; 🡨🡪no statistically significant group difference, NR not reported

[ref.] reference number; nT number of included RCTs; nW number of included women, PTB Preterm birth less than 37 or 32-34 weeks of gestation, LBW low birthweight, SGA small for gestational age, NICU neonatal intensive care unit (admission); Stillb. stillbirth, Miscar. Miscarriage; p.death perinatal death; IUGR intra uterine growth restriction; HIV human immunodeficiency virus

| **3f. Psychosocial interventions and alternative models of care** | | | | | | | | | | | | | | |
| --- | --- | --- | --- | --- | --- | --- | --- | --- | --- | --- | --- | --- | --- | --- |
|  |  | **Primary outcome** | | | | |  | **Secondary outcomes** | | | | | | |
| **First author, year [ref.]** | **Comparison (population)** | **nT** | **nW** | **Statistical method** | **Effect size** | **PTB <37** |  | **PTB <32-34** | **LBW** | **SGA** | **NICU** | **Stillb.** | **Miscar./ perinat.death** | **Side effects** |
| Hodnett 2010 [53] | Additional support versus usual care during at-risk pregnancy (women judged to be at risk of having preterm or growth restricted babies) | 11 | 10 429 | Risk Ratio (M-H, Fixed, 95% CI) | 0.92 [0.83, 1.01] | 🡨🡪 |  | NR | 🡨🡪 | NR | NR | 🡨🡪 | NR | NR |
| Whitworth 2011 [54] | Specialised antenatal care versus routine care (singleton pregnancies at high risk of PTB) | 3 | 3 400 | Risk Ratio (M-H, Fixed, 95% CI) | 0.85 [0.7, 1.03] | 🡨🡪 |  | 🡨🡪 | NR | NR | 🡨🡪 | NR | 🡨🡪 | NR |
| Dowswell 2010 [55] | Reduced number of antenatal care visits/goal oriented versus standard antenatal care visits (women at low risk of developing complications during pregnancy/ labour) | 7 | 60 724 | Risk Ratio (Fixed, 95% CI) | 1.02 [0.94, 1.11] | 🡨🡪 |  | NR | 🡨🡪 | 🡨🡪 | 🡨🡪 | NR | **🡩** | NR |
| Hatem 2009 [56] | Midwife-led versus other models of care for childbearing women and their infants (women classified as low and mixed risk of complications) | 5 | 7 516 | Risk Ratio (M-H, Fixed, 95% CI) | 0.87 [0.73, 1.04] | 🡨🡪 |  | NR | 🡨🡪 | NR | 🡨🡪 | NR | NR | NR |

Group differences: **🡩**statistically significant increase; **🡫**statistically significant reduction; 🡨🡪no statistically significant group difference, NR not reported

[ref.] reference number; nT number of included RCTs; nW number of included women, PTB Preterm birth less than 37 or 32-34 weeks of gestation, LBW low birthweight, SGA small for gestational age, NICU neonatal intensive care unit (admission); Stillb. stillbirth, Miscar. Miscarriage; p.death perinatal death

| **3g. Prevention and management of other morbidities** | | | | | | | | | | | | | | |
| --- | --- | --- | --- | --- | --- | --- | --- | --- | --- | --- | --- | --- | --- | --- |
|  |  | **Primary outcome** | | | | |  | **Secondary outcomes** | | | | | | |
| **First author, year [ref.]** | **Comparison (population)** | **nT** | **nW** | **Statistical method** | **Effect size** | **PTB <37** |  | **PTB <32-34** | **LBW** | **SGA** | **NICU** | **Stillb.** | **Miscar./ p.death** | **Side effects** |
| Kramer 2010 [59] | Increase in exercise in sedentary women (healthy women) | 3 | 111 | Risk Ratio (M-H, Fixed, 95% CI) | 1.82 [0.35, 9.57] | 🡨🡪 |  | NR | NR | NE | NR | NE | NR | NR |
| Kramer 2010 [59] | Increase in exercise in overweight women (healthy women) | 1 | 72 | Risk Ratio (M-H, Fixed, 95% CI) | 1.89 [0.18, 19.95] | 🡨🡪 |  | NR | NR | NR | NR | NR | NR | NR |
| Kramer 2010 [59] | Reduction in exercise in physically fit women (healthy women) | 1 | 61 | Risk Ratio (M-H, Fixed, 95% CI) | 1.18 [0.08, 17.99] | 🡨🡪 |  | NR | NR | NR | NR | NR | NR | NR |
| Muktabhant 2012 [60] | Regular weight measurement versus standard care (general population) | 1 | 235 | Risk Ratio (M-H, Fixed, 95% CI) | 0.67 [0.15, 2.93] | 🡨🡪 |  | NR | 🡨🡪 | 🡨🡪 | NR | NR | NR | NR |
| Lumley 2009 [58] | Interventions for smoking cessation in pregnancy versus control ’usual care’ (all women) | 14 | 11 930 | Risk Ratio (M-H, Random, 95% CI) | **0.86 [0.74, 0.98]** | **🡫** |  | NR | **🡫** | NR | 🡨🡪 | 🡨🡪 | 🡨🡪 | NR |
| Dodd 2010 [57] | Heparin alone or with other medication versus no treatment (women considered at risk of placental dysfunction) | 3 | 237 | Risk Ratio (IV, Fixed, 95% CI) | 0.64 [0.41, 1.01] | 🡨🡪 |  | 🡨🡪 | 🡨🡪 | **🡫** | NR | NR | 🡨🡪 | NE |
| Reid 2010 [61] | Levothyroxine vs. no treatment (women with a diagnosis of hypothyroidism, subclinical hypothyroidism or isolated maternal hypothyroxinaemia) | 1 | 105 | Risk Ratio (M-H, Fixed, 95% CI) | **0.28 [0.1, 0.8]** | **🡫** |  | NR | NR | NR | NR | NR | 🡨🡪 | NR |
| Reid 2010 [61] | Selenomethionine vs. placebo (women with a diagnosis of hypothyroidism, subclinical hypothyroidism or isolated maternal hypothyroxinaemia) | 1 | 151 | Risk Ratio (M-H, Fixed, 95% CI) | 0.96 [0.2, 4.61] | 🡨🡪 |  | NR | NR | NR | NR | NR | 🡨🡪 | NR |
| Say 1996 [62] | Flunarizine versus no treatment (women either at high risk or with suspected impaired fetal growth) | 1 | 100 | Peto Odds Ratio (Peto, Fixed, 95% CI) | 0.5 [0.18, 1.4] | 🡨🡪 |  | NR | NR | NR | NR | NR | 🡨🡪 | NR |

Group differences: **🡩**statistically significant increase; **🡫**statistically significant reduction; 🡨🡪no statistically significant group difference, NR not reported

[ref.] reference number; nT number of included RCTs; nW number of included women, PTB Preterm birth less than 37 or 32-34 weeks of gestation, LBW low birthweight, SGA small for gestational age, NICU neonatal intensive care unit (admission); Stillb. stillbirth, Miscar. Miscarriage; p.death perinatal death
