# Supplementary material for: Antenatal interventions to reduce preterm birth: an overview of Cochrane systematic reviews
Source: BMC Res Notes. 2014 Apr 23;7:265. doi: 10.1186/1756-0500-7-265 (PMC4021758; doi:10.1186/1756-0500-7-265)
Supplement: Additional file 5: Table S4 — Overview of Cochrane reviews that intended to report on PTB < 37 weeks, but were not able to identify any RCTs reporting PTB data. [file 1756-0500-7-265-S5.docx]

**Additional file 5: Table S4: Overview of Cochrane reviews that intended to report on PTB< 37 weeks, but could not report on it due to lack of PTB data from primary research**

**4a. Prevention of PTB or miscarriage and detection of PTB risk**

|  |  | **Review characteristics** | | | | | **Secondary outcomes** | | | | | | |
| --- | --- | --- | --- | --- | --- | --- | --- | --- | --- | --- | --- | --- | --- |
| **First author, year [ref.]** | **Title of Cochrane review** | **Studies published in/ between** | **nStudies** | **nWomen** | **PTB outcome** | **Population** | **PTB <32-34** | **LBW** | **SGA** | **NICU** | **Stillb.** | **Miscar., p.death** | **Side effects** |
| Abdel-Alleem 2010 [64] | Cervical pessary for preventing preterm birth | NA | 0 | NA | primary | Cervical pessary for preventing preterm birth (women with viable fetus in the second trimester of pregnancy and with risk factors for preterm birth due to cervical incompetence) | NA | NA | NA | NA | NA | NA | NA |
| Kaandorp 2009 [74] | Aspirin or anticoagulants for treating recurrent miscarriage in women without antiphospholipid syndrome | 1997-2006 | 2 | 189 | secondary | Aspirin versus placebo (pregnant women with a history of at least two miscarriages without apparent causes other than inherited thrombophilia) | NR | NR | NR | NR | NR | NR | NR |
| Khianman 2012 [75] | Relaxation therapy for preventing and treating preterm labour | 1990-2010 | 11 | 833 | primary | Relaxation versus no treatment/ vs. standard treatment (for women not in PTL) | NR | NR | NR | NR | NR | NR | NR |
| Davey 2011 [65] | Risk scoring systems for predicting preterm birth with the aim of reducing associated adverse outcomes | NA | 0 | NA | primary | Risk scoring systems for predicting preterm birth (all women <37 weeks) | NA | NA | NA | NA | NA | NA | NA |

[ref.] reference number; n number of totally included studies/ women; PTB outcome: Preterm birth was defined as primary, secondary or unspecified outcome in the Cochrane review ;PTB<32-34 Preterm birth less than 32-34 weeks of gestation, LBW low birthweight, SGA small for gestational age, NICU neonatal intensive care unit (admission); Stillb. stillbirth, Miscar. Miscarriage; p.death perinatal death

NR not reported; NA not applicable (no studies included)

**4b. Ultrasound screening (and screening of fetal growth and well-being)**

|  |  | **Review characteristics** | | | | | **Secondary outcomes** | | | | | | |
| --- | --- | --- | --- | --- | --- | --- | --- | --- | --- | --- | --- | --- | --- |
| **First author, year [ref.]** | **Title of Cochrane review** | **Studies published in/ between** | **nStudies** | **nWomen** | **PTB outcome** | **Comparison (Population)** | **PTB <32-34** | **LBW** | **SGA** | **NICU** | **Stillb.** | **Miscar., p.death** | **Side effects** |
| Whitworth 2010 [66] | Ultrasound for fetal assessment in early pregnancy | 1982-2007 | 11 | 37505 | secondary | Routine/revealed versus selective/concealed ultrasound in early pregnancy | NR | 🡨🡪 | 🡨🡪 | 🡨🡪 | NR | 🡨🡪 | NR |
| Alfirevic 2010 [76] | Fetal and umbilical Doppler ultrasound in high-risk pregnancies | 1987-2003 | 18 | 10156 | secondary | Doppler ultrasound versus no Doppler ultrasound (Women with pregnancies considered to be at ’high risk’ for fetal compromise) | 🡨🡪 | NR | NR | 🡨🡪 | 🡨🡪 | **🡫** | NR |
| Stampalija 2010 [77] | Utero-placental Doppler ultrasound for improving pregnancy outcome | 2001-2003 | 2 | 5187 | secondary | Uterine artery Doppler ultrasound versus no Doppler ultrasound, 2nd trimester (all women) | NR | NR | NR | 🡨🡪 | 🡨🡪 | 🡨🡪 | NR |
| Grivell 2010 [78] | Antenatal cardiotocography for fetal assessment | 1982-1999 | 6 | 2105 | secondary | Traditional antenatal CTG versus no antenatal CTG (all women) | NR | NR | NR | 🡨🡪 | NR | 🡨🡪 | NR |
| Mangesi 2007 [79] | Fetal movement counting for assessment of fetal wellbeing | 1989-2004 | 4 | 71370 | secondary | Fetal movement counting vs. no fetal movement counting (all women) | NA | NA | NA | NA | NA | NA | NA |

**4c. Prevention, detection and management of infection**

|  |  | **Review characteristics** | | | | | **Secondary outcomes** | | | | | | |
| --- | --- | --- | --- | --- | --- | --- | --- | --- | --- | --- | --- | --- | --- |
| **First author, year [ref.]** | **Title of Cochrane review** | **Studies published in/ between** | **nStudies** | **nWomen** | **PTB outcome** | **Comparison (Population)** | **PTB <32-34** | **LBW** | **SGA** | **NICU** | **Stillb.** | **Miscar., p.death** | **Side effects** |
| Raynes-Greenow 2004 [80] | Antibiotics for ureaplasma in the vagina in pregnancy | 1987 | 1 | 1071 | primary | Antibiotics versus placebo (women with detection of ureaplasma in the vagina) | NR | 🡨🡪 | NR | NR | NR | NR | 🡨🡪 |

[ref.] reference number; n number of totally included studies/ women; PTB outcome: Preterm birth was defined as primary, secondary or unspecified outcome in the Cochrane review ;PTB<32-34 Preterm birth less than 32-34 weeks of gestation, LBW low birthweight, SGA small for gestational age, NICU neonatal intensive care unit (admission); Stillb. stillbirth, Miscar. Miscarriage; p.death perinatal death

Group differences: **🡫**statistically significant reduction; 🡨🡪no statistically significant group difference; NR not reported; NA not applicable (no studies included)

**4d. Prevention, detection and management of hypertension/ pre-eclampsia and hyperglycaemia/ (gestational) diabetes**

|  |  | **Review characteristics** | | | | | **Secondary outcomes** | | | | | | |
| --- | --- | --- | --- | --- | --- | --- | --- | --- | --- | --- | --- | --- | --- |
| **First author, year [ref.]** | **Title of Cochrane review** | **Studies published in/ between** | **nStudies** | **nWomen** | **PTB outcome** | **Comparison (Population)** | **PTB <32-34** | **LBW** | **SGA** | **NICU** | **Stillb.** | **Miscar., p.death** | **Side effects** |
| Meher 2006 [81] | Garlic for preventing pre-eclampsia and its complications | 2001 | 1 | 100 | primary | Garlic versus placebo/no intervention(all women) | NR | NR | NR | NR | NR | NE | **🡩** |
| Meher 2006 [82] | Rest during pregnancy for preventing pre-eclampsia and its complications in women with nomal blood pressure | 1983 - 1993 | 2 | 106 | primary | Rest alone versus unrestricted activity (women with normal blood pressure) | NR | NR | NR | NR | NR | NR | NR |
| Duley 2010 [83] | Magnesium sulphate and other anticonvulsants for women with pre-eclampsia | 1992-2008 | 15 | 15892 | primary | Magnesium sulphate versus none/placebo (women with pre-eclampsia) | NR | NR | NR | 🡨🡪 | 🡨🡪 | 🡨🡪 | **🡩** |
| Han 2012 [84] | Exercise for pregnant women for preventing gestational diabetes mellitus | 2009-2012 | 5 | 1115 | secondary | Any exercise intervention versus routine care (women without pre-existing type 1 or 2 diabetes) | NR | NR | 🡨🡪 | 🡨🡪 | NR | NR | NR |
| Tieu 2008 [67] | Dietary advice in pregnancy for preventing gestational diabetes mellitus | 1983-2006 | 3 | 107 | secondary | High fibre diet versus normal dietary advice (women without pre-existing type I or type II diabetes mellitus) | NR | NR | NR | NR | NR | NR | NR |
| Tieu 2011 [68] | Screening and subsequent management for gestational diabetes for improving maternal and infant health | 1992 - 2003 | 4 | 3972 | secondary | Risk factor versus universal screening (women without diagnosis of diabetes mellitus) | NR | NR | NR | NR | NR | NR | NR |
| Tieu 2011 [85] | Oral anti-diabetic agents for women with pre-existing diabetes mellitus/impaired glucose tolerance or previous gestational diabetes mellitus | NA | 0 | NA | secondary | Oral anti-diabetic agents vs. none (women with diabetes) | NA | NA | NA | NA | NA | NA | NA |
| Ceysens 2006 [86] | Exercise for diabetic pregnant women | 1989-2004 | 4 | 114 | secondary | Exercise and diet versus diet alone (women with diabetes) | NR | NR | NR | NR | NE | NR | NR |

[ref.] reference number; n number of totally included studies/ women; PTB outcome: Preterm birth was defined as primary, secondary or unspecified outcome in the Cochrane review ;PTB<32-34 Preterm birth less than 32-34 weeks of gestation, LBW low birthweight, SGA small for gestational age, NICU neonatal intensive care unit (admission); Stillb. stillbirth, Miscar. Miscarriage; p.death perinatal death

Group differences: **🡩**statistically significant increase; 🡨🡪no statistically significant group difference; NR not reported; NA not applicable (no studies included); NE not estimable

**4e. Nutritional supplements and dietary interventions**

|  |  | **Review characteristics** | | | | | **Secondary outcomes** | | | | | | |
| --- | --- | --- | --- | --- | --- | --- | --- | --- | --- | --- | --- | --- | --- |
| **First author, year [ref.]** | **Title of Cochrane review** | **Studies published in/ between** | **nStudies** | **nWomen** | **PTB outcome** | **Comparison (Population)** | **PTB <32-34** | **LBW** | **SGA** | **NICU** | **Stillb.** | **Miscar., p.death** | **Side effects** |
| Ballard 2011 [85] | Nutritional advice for improving outcomes in multiple pregnancies | NA | 0 | NA | primary | nutritional advice (women with multiple pregnancies) | NA | NA | NA | NA | NA | NA | NA |
| Thaver 2006 [86] | Pyridoxine (vitamin B6) supplementation in pregnancy | 1960 - 1984 | 5 | 1646 | unspecified | Pyridoxine (B6) versus control (all women) | NR | NR | NR | NR | NR | NR | NR |
| De-Regil 2012 [87] | Vitamin D supplementation for women during pregnancy | 1980-2008 | 6 | 1023 | primary | Vitamin D alone versus no treatment/placebo (all women) | NR | 🡨🡪 | NR | NR | 🡨🡪 | NR | 🡨🡪 |

**4f. Psychosocial interventions and alternative models of care**

|  |  | **Review characteristics** | | | | | **Secondary outcomes** | | | | | | |
| --- | --- | --- | --- | --- | --- | --- | --- | --- | --- | --- | --- | --- | --- |
| **First author, year [ref.]** | **Title of Cochrane review** | **Studies published in/ between** | **nStudies** | **nWomen** | **PTB outcome** | **Comparison (Population)** | **PTB <32-34** | **LBW** | **SGA** | **NICU** | **Stillb.** | **Miscar., p.death** | **Side effects** |
| Lui 2008 [88] | Psychosocial interventions for women enrolled in alcohol treatment during pregnancy | NA | 0 | NA | primary | Psychosocial interventions verus none (women enrolled in alcohol treatment) | NA | NA | NA | NA | NA | NA | NA |
| Terplan 2007 [89] | Psychosocial interventions for pregnant women in outpatient illicit drug treatment programs compared to other interventions | 1995-2004 | 9 | 546 | primary | Any psychosocial interventions versus control (women enrolled in illicit drug treatment programs) | NR | NR | NR | NR | NR | NR | NR |
| Dodd 2012 [90] | Specialised antenatal clinics for women with a multiple pregnancy for improving maternal and infant outcomes | 2006 | 1 | 162 | secondary | ’Specialised’ antenatal clinic versus ’standard’ care (women with multiple pregnancy) | NR | NR | NR | NR | 🡨🡪 | 🡨🡪 | NR |
| Turnbull 2012 [91] | Home visits during pregnancy and after birth for women with an alcohol or drug problem | 1994-2006 | 7 | 950 | unspecified | Home visits versus no home visits during pregnancy or after delivery (women with an alcohol or drug problem) | NR | NR | NR | NR | NR | NR | NR |

[ref.] reference number; n number of totally included studies/ women; PTB outcome: Preterm birth was defined as primary, secondary or unspecified outcome in the Cochrane review ;PTB<32-34 Preterm birth less than 32-34 weeks of gestation, LBW low birthweight, SGA small for gestational age, NICU neonatal intensive care unit (admission); Stillb. stillbirth, Miscar. Miscarriage; p.death perinatal death

Group differences: 🡨🡪no statistically significant group difference; NR not reported; NA not applicable (no studies included)

**4g. Prevention and management of other morbidities**

|  |  | **Review characteristics** | | | | | **Secondary outcomes** | | | | | | |
| --- | --- | --- | --- | --- | --- | --- | --- | --- | --- | --- | --- | --- | --- |
| **First author, year [ref.]** | **Title of Cochrane review** | **Studies published in/ between** | **nStudies** | **nWomen** | **PTB outcome** | **Comparison (Population)** | **PTB <32-34** | **LBW** | **SGA** | **NICU** | **Stillb.** | **Miscar., p.death** | **Side effects** |
| Marc 2011 [92] | Mind-body interventions during pregnancy for preventing or treating women’s anxiety | 1979-2010 | 8 | 556 | secondary | Imagery versus usual care (all women) | NR | NR | NR | NR | NR | NR | NR |
| Earl 2010 [93] | Interventions for preventing and treating hyperthyroidism in pregnancy | NA | 0 | NA | primary | Interventions for preventing and treating hyperthyroidism in pregnancy versus none (women with hyperthyreoidism) | NA | NA | NA | NA | NA | NA | NA |
| Reveiz 2011 [94] | Treatments for iron-deficiency anaemia in pregnancy | 1965-2010 | 23 | 3198 | secondary | Oral iron/ Intravenous iron versus placebo (women with a diagnosis of anaemia attributed to iron deficiency) | NR | NR | NR | NR | NR | NR | 🡨🡪 |

[ref.] reference number; n number of totally included studies/ women; PTB outcome: Preterm birth was defined as primary, secondary or unspecified outcome in the Cochrane review ;PTB<32-34 Preterm birth less than 32-34 weeks of gestation, LBW low birthweight, SGA small for gestational age, NICU neonatal intensive care unit (admission); Stillb. stillbirth, Miscar. Miscarriage; p.death perinatal death

Group differences: 🡨🡪no statistically significant group difference; NR not reported; NA not applicable (no studies included)

**Supplementary References**

[74] Kaandorp S, Di Nisio M, Goddijn M, Middeldorp S. **Aspirin or anticoagulants for treating recurrent miscarriage in women without antiphospholipid syndrome**. *Cochrane Database of Systematic Reviews* 2009, 1: CD004734.

[75] Khianman B, Pattanittum P, Thinkhamrop J, Lumbiganon P. **Relaxation therapy for preventing and treating preterm labour**. *Cochrane Database of Systematic Reviews* 2012, 8: CD007426.

[76] Alfirevic Z, Stampalija T, Gyte GM. **Fetal and umbilical Doppler ultrasound in high-risk pregnancies**. *Cochrane Database of Systematic Reviews* 2010, 1: CD007529.

[77] Stampalija T, Gyte Gillian ML, Alfirevic Z. **Utero-placental Doppler ultrasound for improving pregnancy outcome**. *Cochrane Database of Systematic Reviews* 2010, 9: CD008363.

[78] Grivell RM, Alfirevic Z, Gyte GM, Devane D. **Antenatal cardiotocography for fetal assessment**. *Cochrane Database of Systematic Reviews* 2010, 1: CD008363.

[79] Mangesi L, Hofmeyr GJ, Smith V. **Fetal movement counting for assessment of fetal wellbeing**. *Cochrane Database of Systematic Reviews* 2007, 1: CD004909.

[80] Raynes-Greenow CH, Roberts CL, Bell JC, Peat B, Gilbert GL. **Antibiotics for ureaplasma in the vagina in pregnancy**. *Cochrane Database of Systematic Reviews* 2004, 1: CD003767.

[81] Meher S, Duley L. **Garlic for preventing pre-eclampsia and its complications**. *Cochrane Database of Systematic Reviews* 2006, 3: CD006065.

[82] Meher S, Duley L. **Rest during pregnancy for preventing pre-eclampsia and its complications in women with normal blood pressure**. *Cochrane Database of Systematic Reviews.* 2006, 2: CD005939.

[83] Duley L, Gülmezoglu AM, Henderson-Smart David J, Chou D. **Magnesium sulphate and other anticonvulsants for women with pre-eclampsia**. *Cochrane Database of Systematic Reviews* 2010, 11: CD000025.

[84] Han S, Middleton P, Crowther Caroline A. **Exercise for pregnant women for preventing gestational diabetes mellitus**. *Cochrane Database of Systematic Reviews* 2012, 7: CD009021.

[85] Tieu J, Coat S, Hague W, Middleton P. **Oral anti-diabetic agents for women with pre-existing diabetes mellitus/impaired glucose tolerance or previous gestational diabetes mellitus**. *Cochrane Database of Systematic Reviews* 2011, 10: CD007724.

[86] Ceysens G, Rouiller D, Boulvain M. **Exercise for diabetic pregnant women**. *Cochrane Database of Systematic Reviews* 2006, 3: CD004225.

[87] Ballard CK, Bricker L, Reed K, Wood L, Neilson JP. **Nutritional advice for improving outcomes in multiple pregnancies**. *Cochrane Database of Systematic Reviews* 2011, 6: CD008867.

[88] Thaver D, Saeed Muhammad A, Bhutta Zulfiqar A. **Pyridoxine (vitamin B6) supplementation in pregnancy**. *Cochrane Database of Systematic Reviews* 2006, 2: CD000179.

[89] De-Regil Luz M, Palacios C, Ansary A, Kulier R, Peña-Rosas Juan P. **Vitamin D supplementation for women during pregnancy**. *Cochrane Database of Systematic Reviews* 2012, 2: CD008873.

[90] Lui S, Terplan M, Smith Erica J. **Psychosocial interventions for women enrolled in alcohol treatment during pregnancy**. *Cochrane Database of Systematic Reviews* 2008, 3: CD006753.

[91] Terplan M, Lui S. **Psychosocial interventions for pregnant women in outpatient illicit drug treatment programs compared to other interventions**. *Cochrane Database of Systematic Reviews* 2007, 4: CD006037.

[92] Dodd JM, Crowther CA. **Specialised antenatal clinics for women with a multiple pregnancy for improving maternal and infant outcomes**. *Cochrane Database of Systematic Reviews* 2012, 8: CD005300.

[93] Turnbull C, Osborn DA. **Home visits during pregnancy and after birth for women with an alcohol or drug problem**. *Cochrane Database of Systematic Reviews* 2012, 1: CD004456.

[94] Marc I, Toureche N, Ernst E, Hodnett ED, Blanchet C, Dodin S, et al. **Mind-body interventions during pregnancy for preventing or treating women's anxiety**. *Cochrane Database of Systematic Reviews* 2011, 7: CD007559.

[95] Earl R, Crowther CA, Middleton P**. Interventions for preventing and treating hyperthyroidism in pregnancy**. *Cochrane Database of Systematic Reviews* 2010, 9: CD008633.

[96] Reveiz L, Gyte GML, Cuervo LG, Casasbuenas A. **Treatments for iron-deficiency anaemia in pregnancy**. *Cochrane Database of Systematic Reviews* 2011, 10: CD003094.
